# A Programmable Braided Silk‐Glass Fiber Ligament Promoting Rapid Motoric Recovery

**DOI:** 10.1002/advs.78414

**Published:** 2026-10-29

**Authors:** Changbo Lu, Daming Liu, Haoxin Zhang, Zhiwei Qiao, Qiuming Gao, Jia Meng, Hongyin Yue, Songkai Li, Shuo Zhang, Tao Yuan, Yun Xue, Hui Jiang, Zhenguo Liu, Yibo Wang, Nirong Bao, Yuli Chen, Xiaojiang Yang

**Affiliations:** ^1^ Department of Orthopedics 940th Hospital Lanzhou Gansu China; ^2^ Department of Orthopedics Tangdu Hospital Shaanxi Province China; ^3^ Institute of Solid Mechanics Beihang University Beijing China; ^4^ School of Electronic Information Engineering Beihang University Beijing China; ^5^ Nanjing Fiberglass Research & Design Institute Co., Ltd Jiangsu China; ^6^ Department of Orthopedics, Nanjing Jinling Hospital, Affiliated Hospital of Medical School Nanjing University Jiangsu China; ^7^ Beijing 3D Braiding Co. Ltd Beijing China; ^8^ Nanjing University Institute of Artificial Intelligence Biomedicine Jiangsu China

**Keywords:** ligament tissue engineering, osseoligamentous integration, programmable braiding architecture, soft‐hard tissue interfaces

## Abstract

Anterior cruciate ligament (ACL) reconstruction and related soft‐hard tissue repairs remain challenged by grafts that either lack the characteristic “J‐shaped” stress–strain profile or sacrifice compliance for stiffness while also offering poor biological integration. Here, we report a silk‐glass fiber (SF‐GF) hybrid ligament fabricated by scalable three‐dimensional braiding, combining industrially available glass fibers with biocompatible silk. By tuning the braiding angle ($15^{\circ }$–$45^{\circ }$), the grafts exhibit programmable nonlinear mechanics and viscoelasticity. This design closely replicates the strain‐stiffening response and long‐term creep resistance of native ligaments. Silk‐rich domains promote osseoligamentous integration and osteogenic differentiation, while glass fibers provide high strength and bases for biomimetic mineralization. In rabbit ACL reconstruction, the angle‐optimized SF‐GFs facilitated prompt tendon‐bone healing and restored functional jumping ability within 4 weeks. Finite element modeling rationalizes the strain‐dependent load transfer from silk to glass fibers, underlying the characteristic “J‐shaped” response. Importantly, this material‐structure co‐design leverages mass‐producible components and a manufacturable braiding process, facilitating translational potentials beyond ACL reconstruction toward diverse soft‐hard tissue repairs.

## Graphical abstract

A scalable three‐dimensional braiding strategy integrates silk and glass fibers into customizable artificial ligaments with programmable “J‐shaped” nonlinear mechanics. The silk‐rich architecture supports osseoligamentous integration, while glass fibers provide mechanical reinforcement and biomimetic mineralization cues, enabling strain‐adaptive load transfer, accelerated tendon‐bone healing, and functional motor recovery within 4 weeks after rabbit ACL reconstruction.

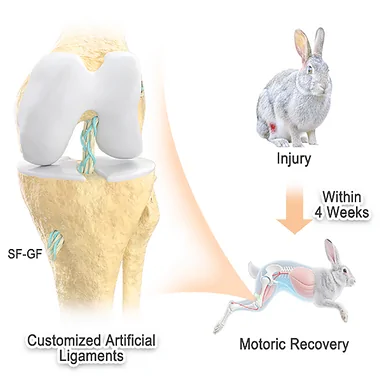

## 1 Introduction

The anterior cruciate ligament (ACL) is a highly specialized fibrous tissue that orchestrates the stability and mobility of the knee joint through aligned collagen fibers anchored in bone. Yet this vital structure is unable to self‐regenerate [1]. With over 650 000 worldwide ACL ruptures annually [2, 3], ACL reconstruction remains a considerable unmet medical need. Currently, biological autografts and allografts used in ACL reconstruction include the hamstring tendon and patellar tendon. Although the most common choice for surgeons [4], they suffer from drawbacks such as donor site morbidity and mechanical laxity at harvest site. Synthetic alternatives, such as the LARS poly(ethylene terephthalate) (PET) ligament, were designed to replace biological grafts in clinical trials for their abundance and high chemical stability [5, 6]. However, the commercialized ligaments fail to replicate the ACL's nonlinear elasticity and are intrinsically bioinert to induce graft‐bone healing, which may lead to long‐term implant failure [7, 8].

The hierarchical structure of native ligaments provides a biomimetic blueprint. Human ligaments are typically assembled from collagen fibril bundles hinged on two brilliantly evolved features [9, 10]. The crimped collagen fibrils that uncurl like springs during initial stretch (the “toe region”), cushioning low‐stress movements [11]. Helical fibrils are present where the tissue must resist multidirectional or unpredictable loading [12, 13, 14]. Crucially, as fundamentally established by Fung [15], the mechanical behavior of such soft tissues is inherently viscoelastic. The shape of their nonlinear, “J‐shaped” stress–strain curve is highly time‐dependent and significantly influenced by the strain rate at which the multidirectional loads are applied [16].Moreover, at the anchoring site of the ligament, the graded enthesis exhibits a smooth transition from soft non‐mineralized fibrocartilage to stiff bone, enabling stress distribution via a distinctive toe‐linear mechanical response [17]. Consequently, replicating such a complex hierarchical architecture presents two main challenges: capturing these dynamic mechanobiological behaviors (i.e., nonlinear elasticity and strain‐rate dependence) and achieving robust biological integration between ligament and bone. To promote ligament‐bone integration, tissue engineering has emerged as a promising strategy. Some have opted for the design of scaffolds that are loaded with bioactive components such as growth factors [18, 19, 20], or structural proteins [21]. However, such bioactive enhancements in tissue engineering add complexity and have demonstrated more compelling therapeutic effects [22, 23]. Silk fibroin (SF) may be particularly appealing fiber material due to its broad sources and exceptional biocompatibility with surrounding tissue. Nevertheless, the biodegradability property of SF may lead to losing mechanical strength too rapidly for ACL reconstruction demands [24, 25, 26]. To address the multifaceted requirements of biocompatibility, mechanical strength, and osteoinductive potential in bone—graft integration, we propose a silk‐glass fiber (SF‐GF) hybrid fibrous scaffold strategy that combines the high tensile strength of glass fibers with the elasticity and bioactivity of silk fibers. In this design, bioactive silk fibroin, mimicking the crimp‐mediated compliance of the native ligament toe region, is helically braided into glass fiber lattices that provide linear‐region stiffness and bases for biomimetic mineralization. Beyond mere biomimicry, the mechanical stiffness and dynamic mechanical responses of the scaffold can be precisely tuned by adjusting the silk‐to‐glass ratio and the braiding angle, enabling patient‐specific customization. This represents a first attempt for synthetic ligament design and offers translational promise. We hypothesize that this biphasic, biomimetic architecture not only replicates native ACL mechanics but also promotes accelerated osseoligamentous integration, marking a shift from static implants toward dynamic, regenerative solutions (Figure 1).

**FIGURE 1:**
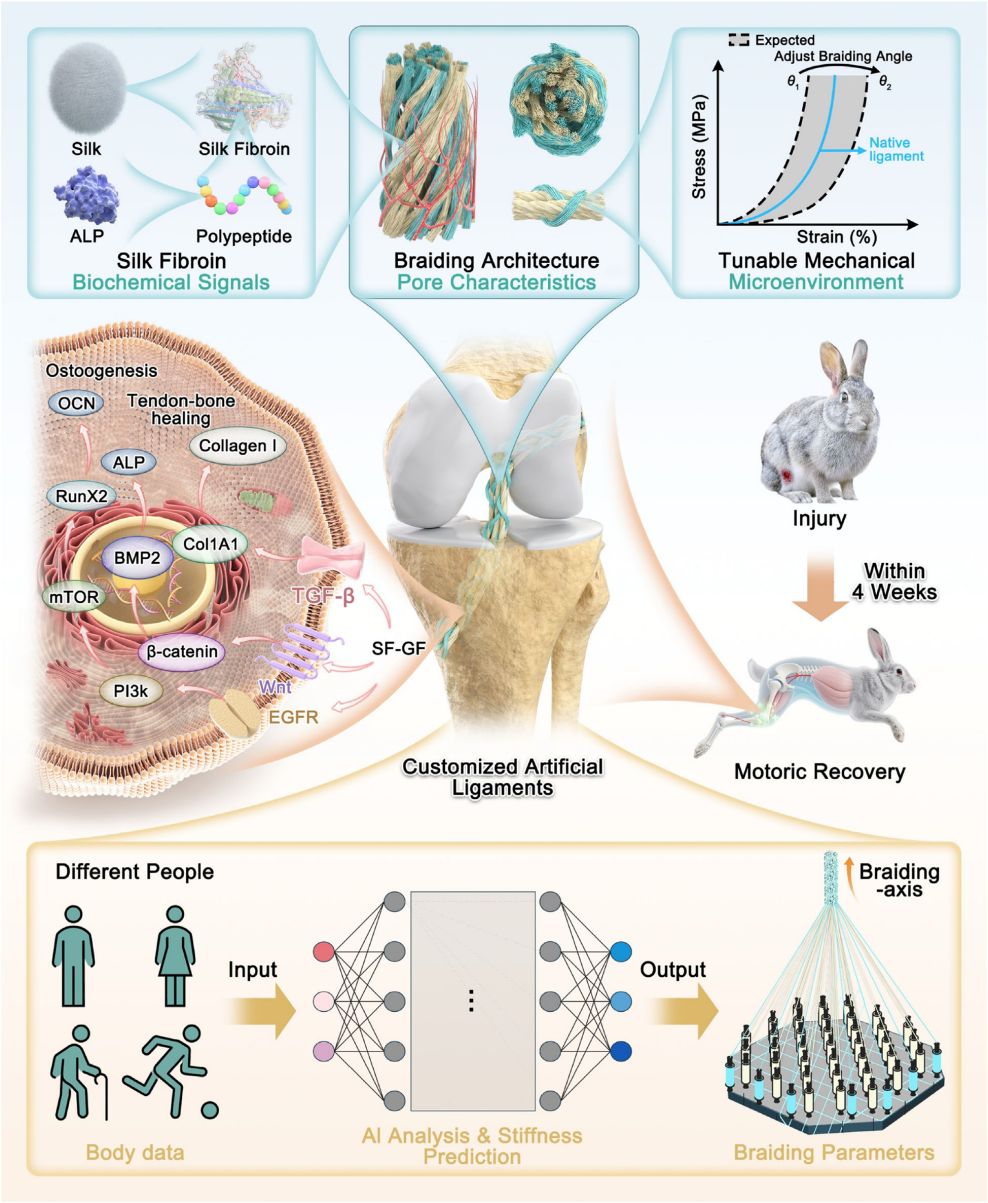
Mechanobiological advantages and future prospects of SF‐GF grafts.

## 2 Results

### 2.1 Structural Characterization and Programmable Mechanical Design of SF‐GF Scaffolds

A novel braiding system (3DB‐R100/1C1, China, Figure 2a) was employed to fabricate a hybrid artificial ligament using a three‐dimensional five‐directional braiding technique, in which the weft yarns are arranged into a distinctive mesh‐like architecture (Figure 2b). The ligament, denoted as SF‐GF, consists of 51 glass fibers as weft yarns and 37×2 tussah silk fibers as axial yarns, where the silk fibers were braided in a two‐in‐one configuration within each carrier. The unique braiding architecture produced by this system generates an architected weft–yarn network (Figure 3a), which enables originally linear‐elastic fibers to exhibit a characteristic “J‐shaped” stress–strain response along the axial direction. Owing to the wrapping and interlacing of the weft yarns, the effective stiffness of the braided ligament is lower than that of the constituent filaments (Figure 3c,d), facilitating nonlinear deformation.

**FIGURE 2:**
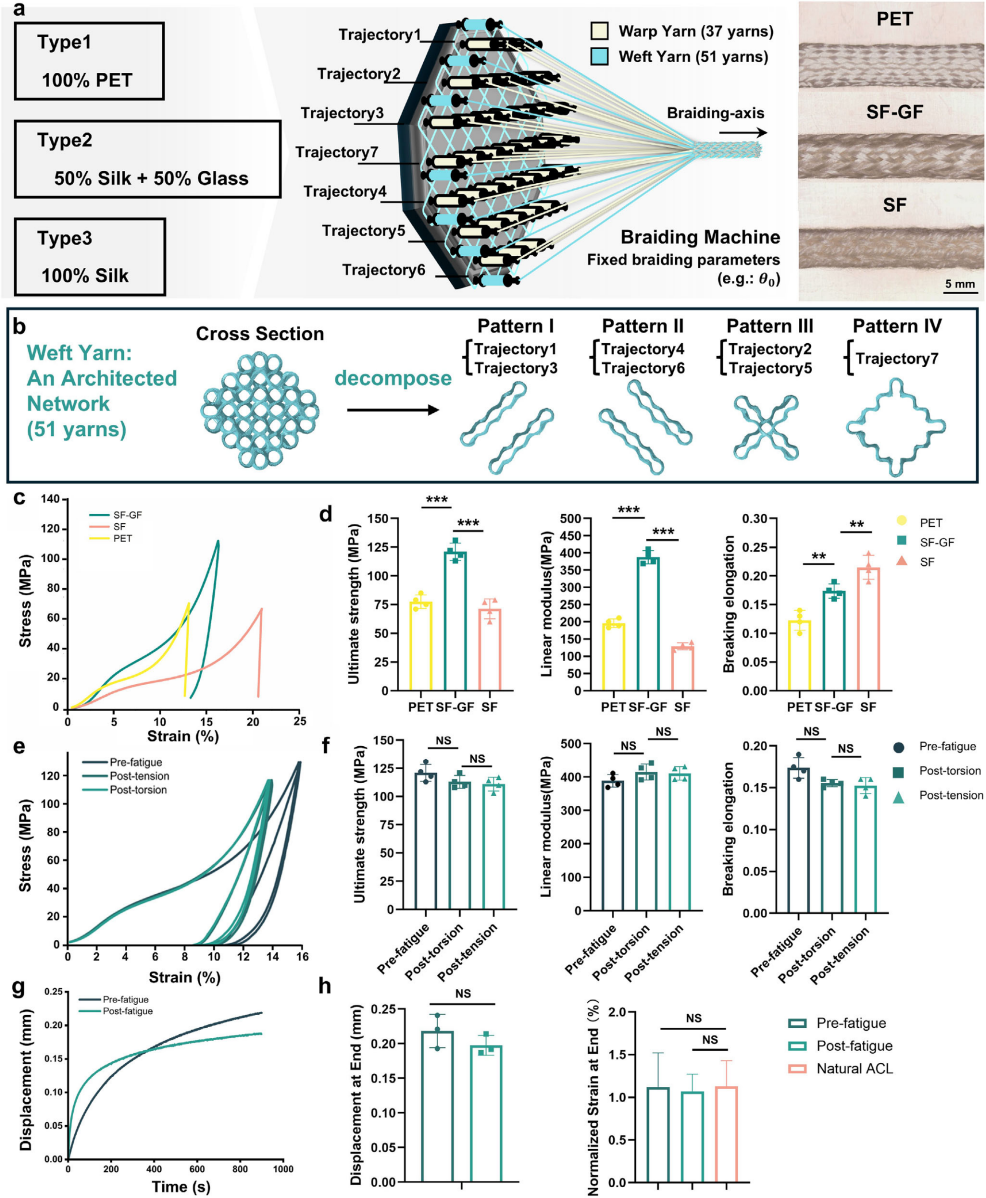
Composite mechanical properties of ligaments with different fiber components. (a) Three‐dimensional five‐directional braiding process of the hybrid artificial ligament. (b) The weft yarn architecture consists of four distinct patterns, each formed by specific combinations of trajectory groups. (c) Stress–strain curves of PET, SF, and SF‐GF ligaments. (d) Comparative mechanical properties: UTS, modulus, failure strain. (e) Tensile properties of SF‐GF grafts before/after $5\times 10^{6}$ cyclic fatigue under tensile and torsional loading. (f) Fatigue‐induced changes in ultimate tensile strength (UTS) and modulus (n=4, mean ± s.d.; NS, not significant, *p<0.05, **p<0.01, ***p<0.001, one‐way ANOVA with Tukey's post hoc test). (g) Creep behaviors: time vs. displacement of SF‐GF grafts. (h) Normalized strain at the end of loading (constant load: 250 N).

**FIGURE 3:**
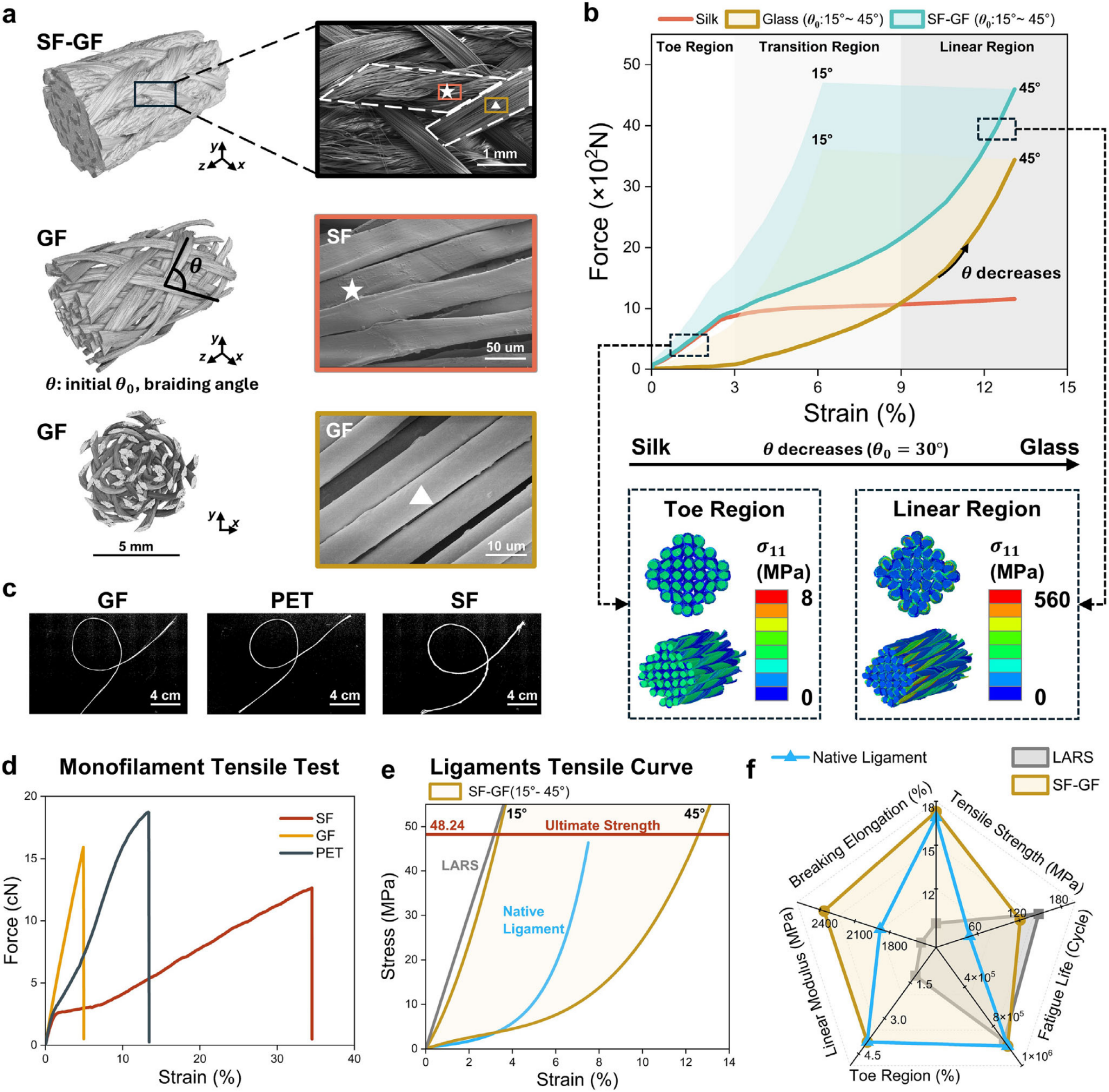
Mechanical behavior of SF‐GF scaffolds. (a) Three‐dimensional reconstruction of the braided network extracted from the SF‐GF grafts and a cross‐sectional scanning electron microscopy (SEM) image of the SF‐GF hybrid ligament. Pentagons indicate intralamellar SF bundles; triangles indicate GF bundles. (b) Representative stress–strain curve of the SF‐GF scaffold under tensile loading, illustrating its characteristic nonlinear response mechanism. (c) Photographs of continuous glass fiber (GF), polyethylene terephthalate (PET), and silk fibroin (SF) monofilaments. (d) Representative specific stress–strain curves of SF and GF monofilaments under quasi‐static tension. (e) Mechanical regulation range of SF‐GF ligament vs. LARS and native ligament. (f) Comparison of the mechanical parameters of the SF‐GF ligament vs. LARS and native ligament.

To identify the optimal reinforcement strategy, glass fibers, PET fibers (the raw material of the widely used commercial LARS ligament), and silk fibers were braided into artificial ligaments using an identical processing route (braiding angle of $30^{\circ }$). SF‐GF exhibited higher strength than both SF and PET controls (Figure 2c), achieving a pre‐fatigue ultimate strength of 121 ± 7.6 MPa (Figure 2d) together with an expanded working‐strain window (Breaking elongation = 0.173, Figure 2d). In contrast, PET‐based ligaments showed substantially lower stiffness and strength when used as braided yarns, with tensile strength only marginally meeting the requirements of native human ligaments [27].

The fatigue performance of SF‐GF ligaments was further assessed. After five million loading cycles, the ligaments retained large strain amplitudes (15.1% in tension and 15.4% in torsion) and high post‐fatigue strengths (111–113 MPa) (Figure 2e,f), confirming their superior strength, extensibility, and long‐term durability. It should be noted that glass fibers may release micro‐scale debris during the dynamic loading (Figure S8b). Under constant physiological loading, SF‐GF ligaments exhibited a reduced creep rate post‐fatigue compared to the pre‐fatigue state, likely due to cyclic structural compaction (Figure 2g: 1.18% vs. 1.07%). Importantly, both pre‐ and post‐fatigue creep values remained well within the acceptable physiological range for native ligament (Figure 2h: 1.13% ± 0.23%). This robust dimensional stability effectively mitigates continuous plastic elongation, mirroring the viscoelastic stability of biological tendon grafts. As discussed in the Supporting Information, previous studies have reported that glass fiber wear particles can promote apatite layer formation [28, 29], suggesting that their biological impact is limited and may even be beneficial under certain conditions (Figure S8c).

The influence of SF volume fraction on SF‐GF ligament performance was preliminarily assessed using SF contents of 100%, 75%, and 65%. The ligament composed entirely of SF was excluded from further study due to the inherent in vivo degradability of silk. Increasing the glass fiber fraction enhanced structural stiffness. However, the 75% SF ligament exhibited excessive rigidity, which is unfavorable for ACL reconstruction because it requires mechanical compliance under combined loading. Consequently, an SF volume fraction of 65% was selected for subsequent investigations.

The characteristic “J‐shaped” stress–strain behavior of SF‐GF scaffolds, composed of silk fibers as axial yarns and glass fibers as braided weft yarns, arises from progressive load transfer within the three‐dimensional architecture (Figure 3a). At low strain, deformation is dominated by the ductile silk fibers, producing a compliant toe and transition region, whereas increasing strain promotes friction‐mediated load transfer to the stiff glass fiber network, resulting in a rapid stiffness increase (Figure 3b). The braiding angle ($\theta_{0}$) critically regulates this behavior: increasing ($\theta_{0}$) reduces overall stiffness while amplifying the transition region and “J‐shaped” nonlinearity. At small $\theta_{0}\left(15^{\circ }\right)$, a “rope‐binding‐straw” effect emerges, in which glass fibers carry the load from the onset and locally constrain silk fibers, inducing lateral bulging and combined tensile‐shear stresses that may promote fatigue initiation. Compared with the commercial LARS ligament, SF‐GF ligaments more closely replicate the nonlinear mechanical response of native ligaments, while exhibiting an appropriate toe region (Figure 3e,f).

### 2.2 In Vitro Cytocompatibility and Osteogenic Differentiation Mechanisms

#### 2.2.1 SF‐GF Ligament is Biocompatible and Promotes Graft‐Ligament Healing

An appropriate ligament must not only have good biomechanical properties but also have excellent tissue biocompatibility. Hence, to evaluate the biocompatibility of SF‐GF in vitro, L929 cells were cultivated under SF‐GF composite extract (Figure 4a,c and Figure S7). Within one week post cultivation, the proliferation assays revealed that cell viability, reflected in the absorbance values, increased significantly between day 1 and day 3 (p<0.001), and between day 3 and day 7 (p<0.001), in the presence of SF‐GF extracts (Figure 4b). No significant difference in proliferation was observed between cells cultured in standard medium and those exposed to SF‐GF composite extracts.

**FIGURE 4:**
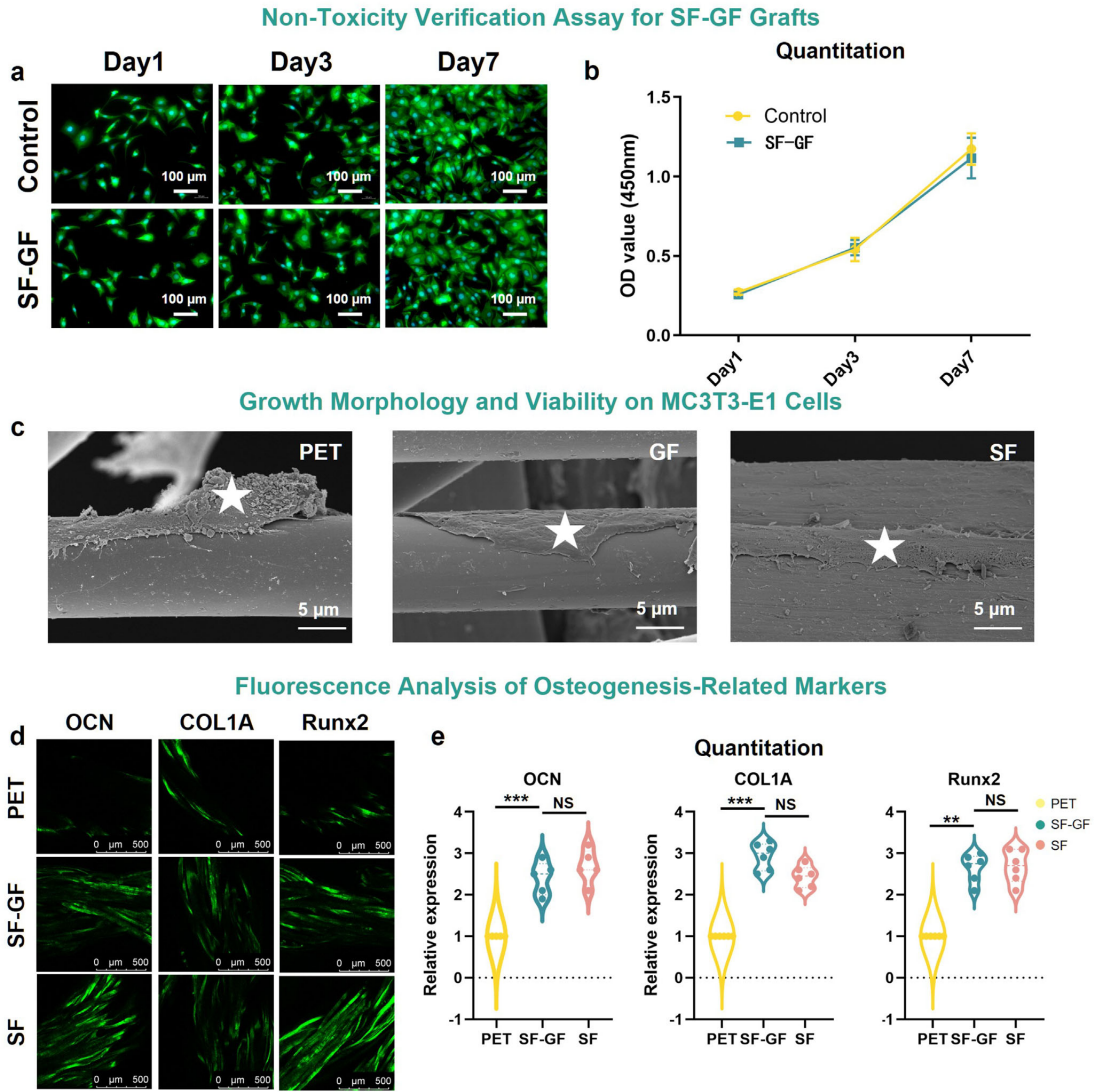
Cytocompatibility and osteogenic response of SF‐GF scaffolds. (a) Proliferation of L929 cells in SF‐GF graft extracts and α‐MEM on days 1, 3, and 7 (blue: nuclei, green: F‐actin). (b) CCK‐8 assay showing no cytotoxicity of SF‐GF extracts (mean ± SD, n=6). (c) SEM images reveal cell (asterisks) attachment and proliferation: cells on SF scaffolds appear well‐spread, while those on GF and PET scaffolds shrink or die. (d) Viability of osteogenic markers (OCN, COL1, Runx2) in MC3T3‐E1 cells cultured on scaffold segments through immunofluorescence staining. (e) Semi‐quantitative fluorescence of osteogenic markers (OCN, COL1, Runx2; n=6, mean ± s.d.; NS not significant, *p<0.05, **p<0.01, ***p<0.001).

To elucidate the molecular mechanisms by which silk fibroin‐based scaffolds enhance osteogenesis, we performed RNA‐seq on MC3T3‐E1 cells cultured on PET (control), SF, and SF‐GF scaffolds (Figure 5a–c). Differential gene expression analysis identified a significant upregulation of pro‐osteogenic transcripts in cells grown on both SF and SF‐GF scaffolds compared to the PET plate and ligament controls (Figure 5d). Notably, the SF scaffolds demonstrated a more pronounced effect, inducing a greater number and a higher fold‐change of genes associated with bone formation, indicating the SF components could activate the osteogenic‐related signaling pathway. Pathway enrichment analysis of the differentially expressed genes (DEGs) revealed that the most significantly activated pathways were the Wnt/β‐catenin and TGF‐β signaling pathways (Figure 5e,f and Figure S2). More specifically, first, comparative transcriptomic analyzes of SF ligament fibers vs. PET ligaments, as well as GF plates vs. PET plates, indicated that the glass fiber components were associated with activation of the TGF‐β signaling pathway. Second, comparison between SF plates and PET plates suggested that the SF components preferentially activated the Wnt signaling pathway. Finally, comparisons between SF ligaments and SF plates revealed that the porous microstructure in the ligament configuration markedly upregulated multiple osteogenic signaling pathways, including the PI3K–Akt, Wnt, and TGF‐β pathways (Figure 5e,f).

**FIGURE 5:**
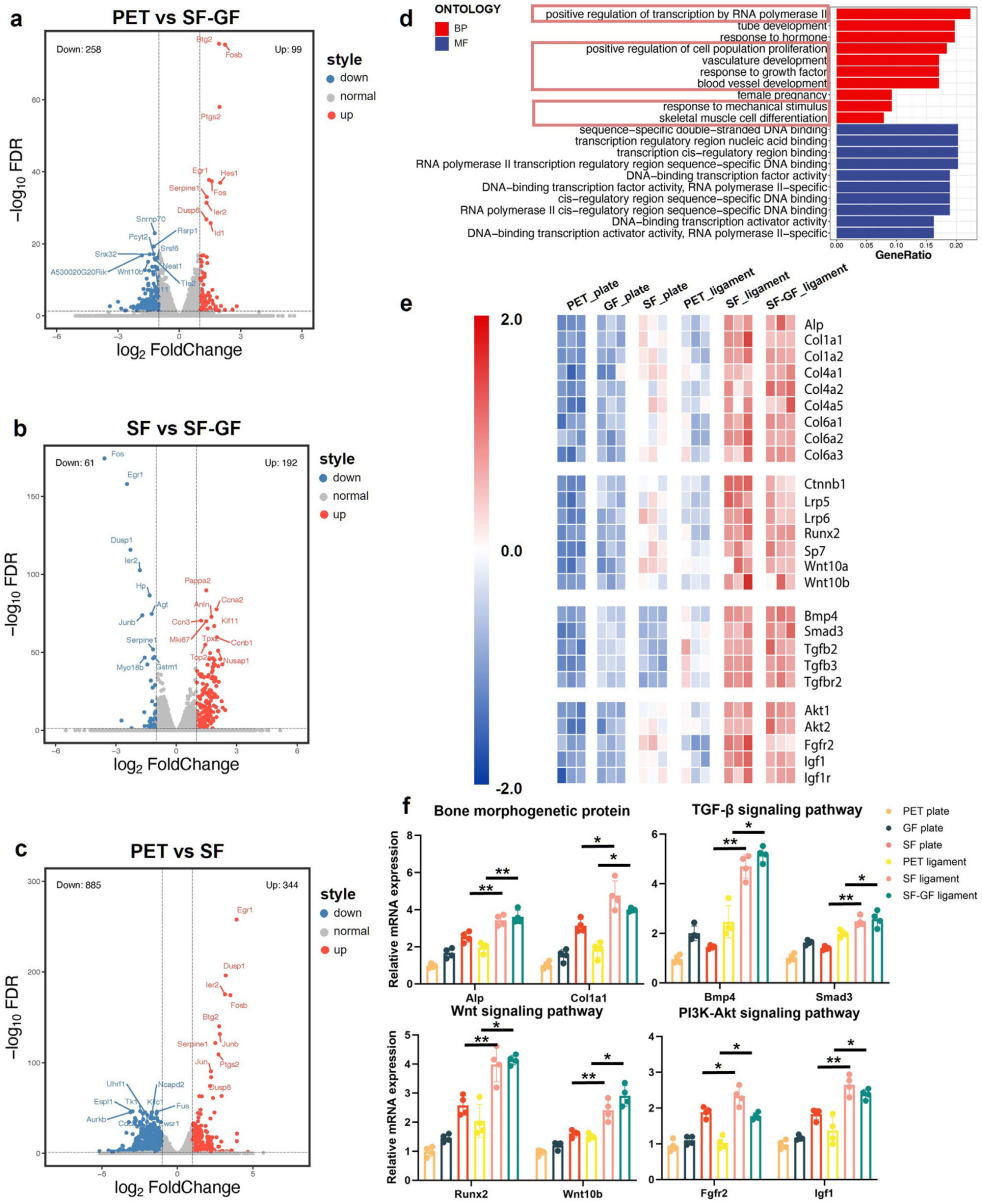
SF‐GF promotes osteogenesis in vitro via signalling pathways. (a–c) Volcano plots of transcriptome on different grafts comparison. (d) GO enrichment based on transcriptomic analysis of MC3T3‐E1 cells shows upregulated osteogenesis‐related signalling pathways in the PET and SF‐GF comparison. (e) Clustered heatmap of pathways of osteogenic/osseoligamentous in PET plate, SF plate, SF‐GF plate, PET ligament, SF ligament and SF‐GF ligament. (f) Expression of osteogenic genes in BMP, Wnt, TGF‐β, PI3K‐Akt (n=4, mean ± s.d.; NS, not significant, *p<0.05, **p<0.01, ***p<0.001).

Complementing the genotypic analysis, the effects of scaffolds on osteogenic protein synthesis were assessed via immunodetection of collagen I (COL1A), osteocalcin (OCN) and Runt‐related transcription factor 2 (Runx2), two late‐stage bone‐specific glycoproteins (Figure 4d and Figure S3). Confocal micrographs (Figure 4e) revealed minimal COL1A, OCN and Runx2 expression in MC3T3‐E1 cells cultured on PET scaffolds after 5 days. In contrast, SF‐GF scaffolds exhibited significantly higher OCN and Runx2 levels compared to PET scaffolds, further confirming superior osteoinductive potential of the SF‐GF and SF scaffolds.

### 2.3 In Vivo Biosafety, Motoric Recovery, and Osseoligamentous Integration

#### 2.3.1 SF‐GF Ligament is Biocompatible In Vivo

Long‐term biosafety of SF‐GF was evaluated through the inflammation score after implantation of the graft in rabbits for 12 weeks. As shown in Figure S8, no signal characteristic of major inflammation can be observed. Wear debris (triangle) implanted subcutaneously is surrounded by unstructured tissue and is limited by a thin layer of flat nucleated cells. Further laboratory inspection revealed that IL‐1 and TNF‐α concentrations varied within normal physiological ranges (n=4 biologically independent experiments for each group).

#### 2.3.2 SF‐GF Promotes Osseointegration In Vivo

To assess the in vivo performance of SF‐GFs, we implanted grafts into a rabbit ACL reconstruction model. Following standard surgical procedures (Figure 6a,b), 3‐mm bone tunnels were drilled into the femur and tibia, through which SF‐GFs were implanted and secured to the host bone (Figure 6b,c). In motor function assessments, open‐field and gait analysis tests were conducted. In the open‐field test, rabbits with ACL reconstruction exhibited a significant increase in movement distance and average speed at 7 days post‐surgery. At 1 month post‐surgery, during autonomous movement, the movement distance of rabbits in the SF‐GF group was significantly greater (Figure S4). Owing to strong interfacial bonding driven by bone regeneration, rabbits implanted with SF‐GFs recovered rapidly, displaying normal gait and free jumping without limping by week 4 after surgery (Figure 6 and Supporting Video). In contrast, control rabbits with untreated ligament rupture failed to complete a jump and displayed limp movement trail (Figure 6d). As expected, no significant changes in the inflammation score of the synovium, liver, and kidney were observed (Figure S1).

**FIGURE 6:**
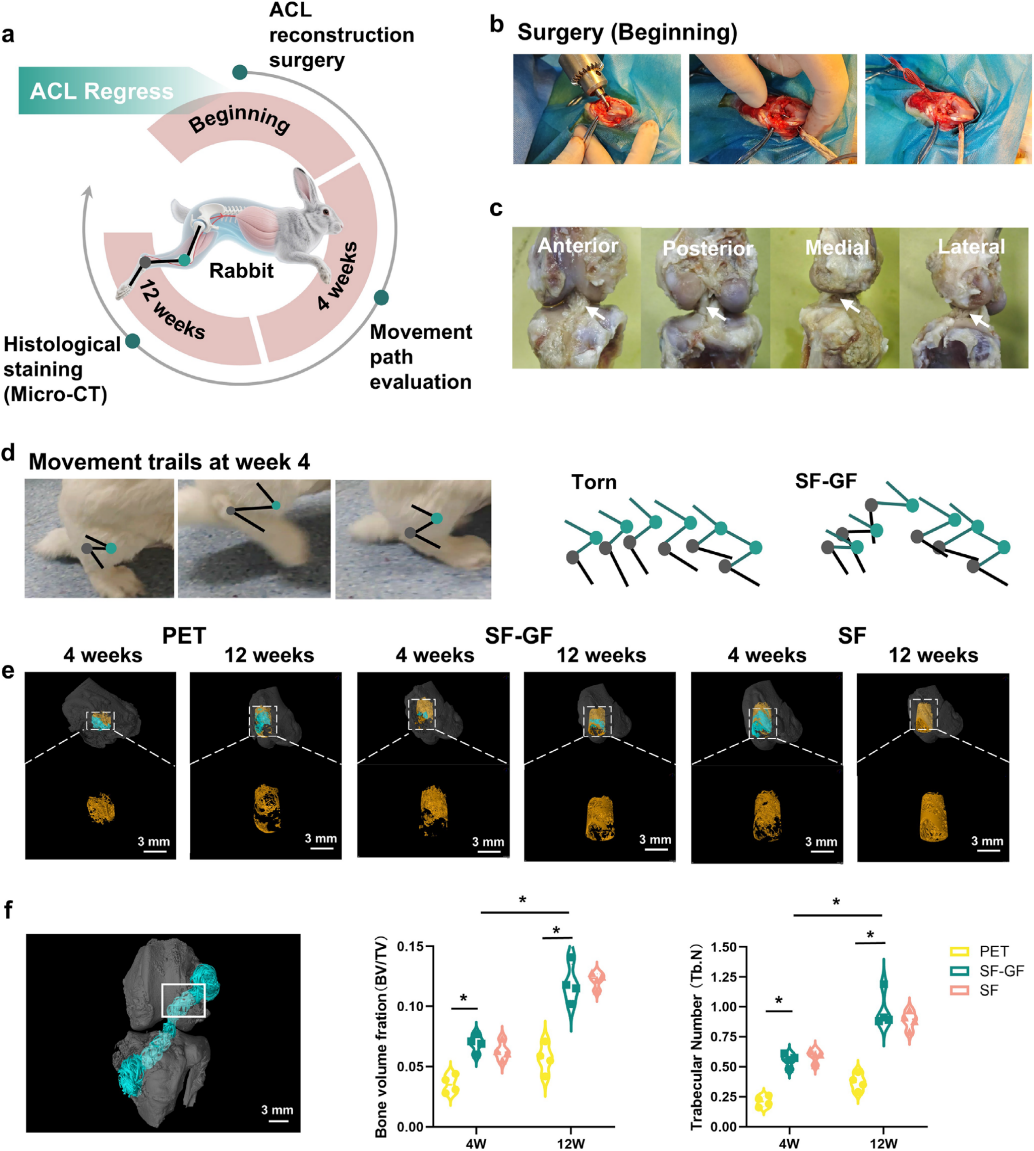
Schematic of animal experiment in rabbit ACL reconstruction model. (a) Rabbit ACL recovery schedule. (b) Surgery schematic using SF‐GF as artificial ligament; graft passed through 3 mm tunnels and fixed by knots. (c) Femur‐graft‐tibia complex at 4 w (white arrowheads: SF‐GF). (d) Knee movement trail showing jump kinematics in SF‐GF vs. ACL‐torn rabbits at 4 w. (e) μCT images of femur ends at 4 and 12 w with PET, SF‐GF and SF grafts. (f) Osteogenic quantification at graft–tunnel interface with PET, SF‐GF and SF grafts (BV/TV: bone volume/ total volume; Tb.N: Trabecular Number; n=4, mean ± s.d.; NS, not significant, *p<0.05, **p<0.01, ***p<0.001).

Microcomputed tomography (μCT) confirmed significant osseointegration within the bone tunnel after SF‐GF implantation (Figure 6e and Figure S5). Bare helical PET fibers of identical size and structure served as controls and showed limited osteointegration. Newly formed bone was observed at the graft‐bone interface in the SF‐GF group (Figure 6e) compared with ACL‐torn rabbits, indicating robust osseointegration. Quantitative μCT analysis demonstrated that bone volume fraction (BV/TV) and bone surface fraction (BS/TV) in the SF‐GF and SF groups were significantly higher than those of PET fibers at both 4 and 12 weeks (Figure 6e,f). For SFs, SF‐GFs, and PETs, BV/TV values were 15.2%, 11.2%, and 4.2% at week 4, increasing to 22.3%, 18.4%, and 5.1% at week 12, respectively. BV/TV and BS/TV in the SF‐based groups were approximately twofold greater than PET controls, confirming the strong osteoinductive capacity of SF‐containing grafts (Figure 6f).

Histological staining (hematoxylin–eosin, H&E; Van Gieson, VG; Masson) revealed no local inflammatory responses (Figure 7a), underscoring the biocompatibility of SF‐GFs. Consistent with μCT and biomechanical findings, regenerated collagen fibers were observed within the SF‐GF scaffolds, integrating progressively with surrounding bone tissue (Figure 7). Collagen deposition at the graft‐bone interface increased from 4 to 12 weeks, with fibers infiltrating scaffold pores by week 12.

**FIGURE 7:**
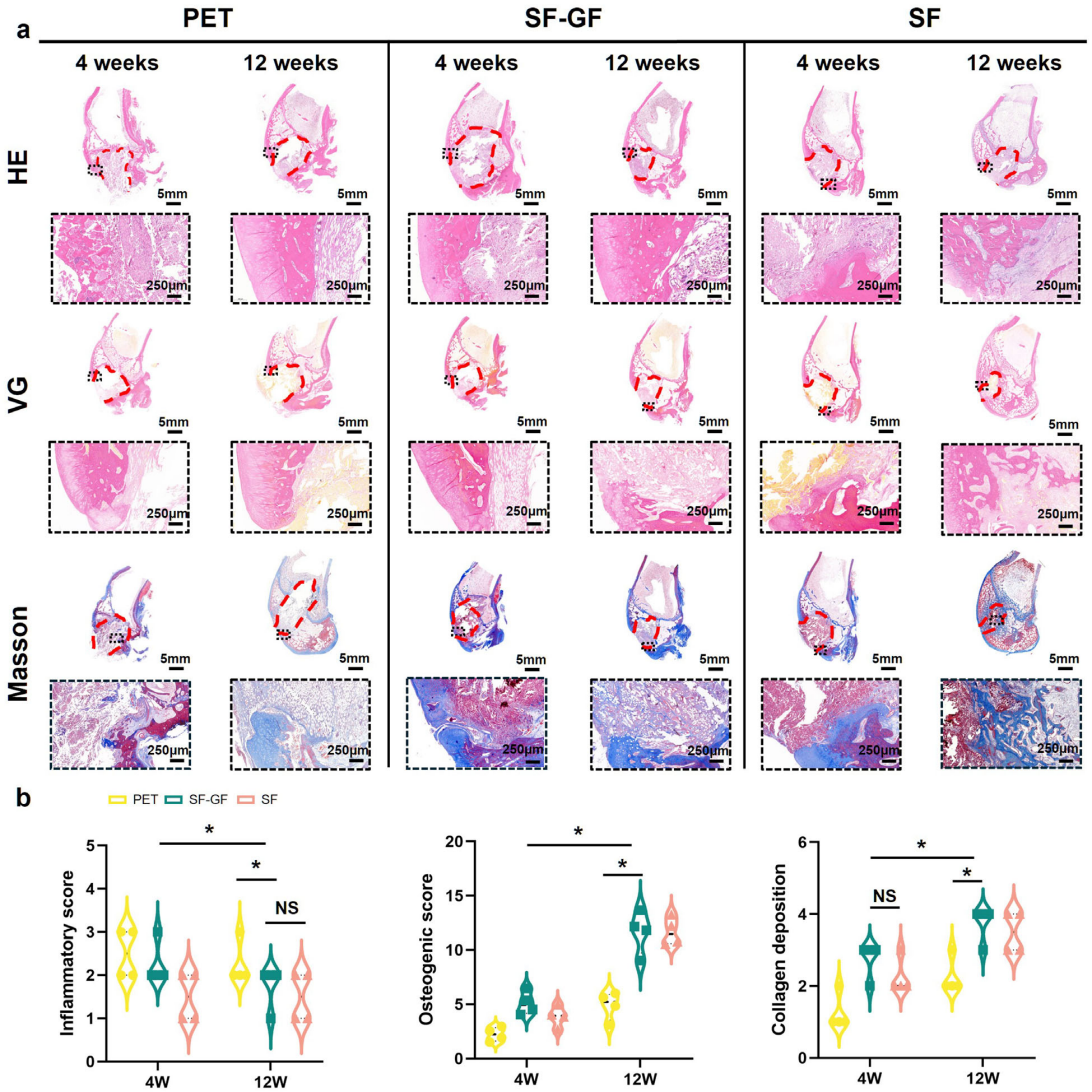
Osteogenic marker expression around SF‐GF grafts. (a) H&E, VG, Masson staining at 4 & 12 w (ACL reconstruction). Dashed lines: graft–tunnel boundary. (b) Histomorphometric scores: inflammation (H&E), bone regeneration (H&E/VG), collagen deposition (n=4, mean ± s.d.; NS, not significant, *p<0.05, **p<0.01, ***p<0.001).

To further elucidate the mechanism, immunofluorescence staining showed that SF‐GFs enhanced osteoblast activity, with markedly elevated Runx2 and OCN expression compared with PET controls (Figure 8). These findings suggest that SF‐GFs may promote osteointegration by activating osteogenesis‐related pathways, including Wnt and TGF‐β signaling [30].

**FIGURE 8:**
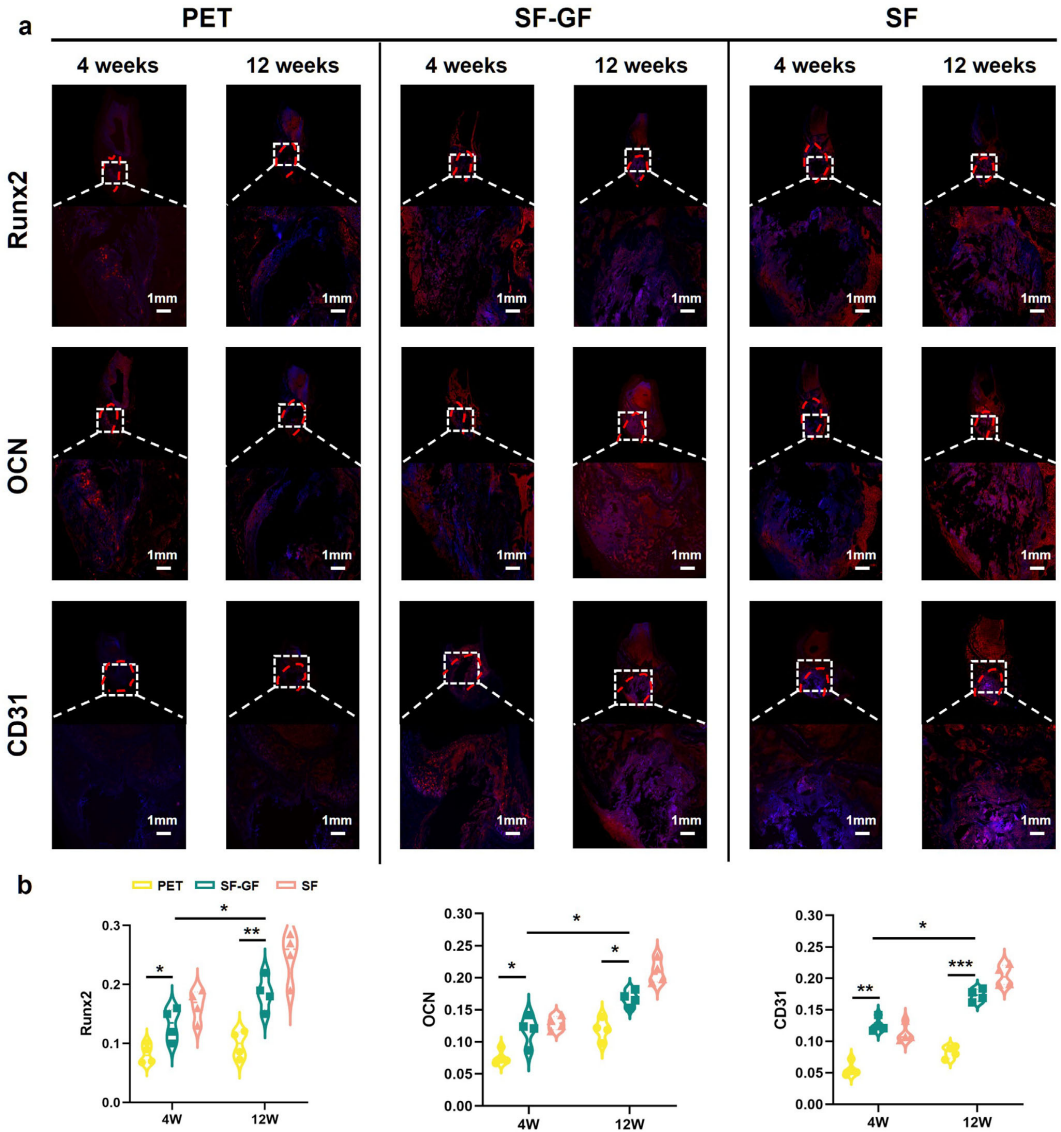
Osteogenic and vascularization marker expression around SF‐GF grafts. (a) Immunofluorescence staining at 4 & 12 w. (b) Runx2, OCN, CD31 intensity (n=4, mean ± s.d.; NS, not significant, *p<0.05, **p<0.01, ***p<0.001).

To directly assess vascularization, we performed immunohistochemical staining for the endothelial cell marker CD31. SF‐GF grafts exhibited a higher density of CD31‐positive blood vessels at the interface compared with PET controls (Figure 8), confirming enhanced vascular ingrowth and superior osteogenic integration.

## 3 Discussion

This study pioneers an architecturally tunable SF‐GF hybrid graft that addresses critical limitations in ACL reconstruction. By integrating bioactive silk fibroin with high‐strength glass fibers through a three‐dimensional angle‐ply braiding strategy, we simultaneously tackle two longstanding challenges in synthetic ligament design: biomechanical mimicry and effective osseointegration.

### 3.1 Soft‐to‐Stiff Hybrid Design Enables Ligament‐Mimetic Mechanics

ACL function relies on crimped collagen fibrils, which impart strain‐dependent stiffness‐initially compliant in the toe region and progressively stiffer with increasing load [31]. Commercial PET grafts fail to reproduce this nonlinear response, and although hybrid PET designs have been proposed to improve strength and fatigue resistance, their biological performance remains uncertain. In contrast, our SF‐GF hybrid graft achieves functional fidelity through precise architectural design. By combining ductile, strain‐hardening silk fibers with stiff glass fibers in a three‐dimensional braided architecture, the scaffold reproduces the biphasic “J‐shaped” stress–strain profile of native ligaments. Silk fibers govern the compliant toe region, analogous to collagen uncrimping, while frictional interactions gradually transfer the dominant load to the glass fibers, which reinforce the construct under higher strains. Finite element analysis further revealed a “rope‐binding‐straw” effect at small braiding angles, highlighting localized stress concentrations where silk is locked by glass. Meanwhile, the hybrid design maintained high extensibility, superior strength, and robust fatigue resistance over millions of cycles.

Crucially, this structural stability extends to the graft's dynamic viscoelastic behaviors. Since time‐dependent elongation (creep) under continuous physiological loading is a primary driver of post‐surgical joint laxity, resistance to continuous plastic deformation is paramount. The SF‐GF grafts exhibited robust creep resistance, with fatigue‐induced structural compaction further slightly reducing the post‐fatigue creep rate, ensuring long‐term dimensional stability and reliable mechanical durability.

Beyond ACL reconstruction, these findings point to a broader design principle. The deliberate integration of soft and stiff fibers enables tunable, strain‐dependent mechanics and provides a generalizable framework for engineering soft‐hard interfaces. This soft‐to‐stiff design strategy not only offers a promising solution for ligament repair but also establishes a versatile paradigm for other musculoskeletal junctions, such as tendon‐to‐bone and cartilage‐to‐bone—where graded mechanics are critical for long‐term function.

### 3.2 Osteogenic Bioactivity Driven by Topographical and Biochemical Cues

The interface between the ACL and bone is a heterotypic gradient tissue, presenting a major challenge for tissue engineering. The incorporation of bioactive components can promote cell adhesion and differentiation. However, their complex fabrication processes and high costs limit widespread clinical application. In contrast, silk fibers, derived from silk cocoons, are capable of providing inherent biochemical signals to guide and influence cells [32, 33, 34]. Moreover, recent studies have emphasized the importance of surface topography and fiber alignment in directing stem cell differentiation toward osteogenic lineages [35, 36].

Microfiber scaffolds with fiber orientations of $90^{\circ }$ and $60^{\circ }$ have been shown to enhance osteogenic differentiation by modulating the YAP pathway and upregulating the expression of Runx2 and OCN [37]. Immunofluorescence staining results in our study validate this phenomenon, revealing a significant upregulation in the expression of osteogenic markers—OCN and Runx2—and the ligament‐related gene Col I [38, 39]. Furthermore, by means of transcriptomic and immunodetection analysis of specific proteins, we identified the Wnt, TGF‐β, and PI3K‐Akt signaling pathways as being involved in the osseoligamentous integration process. SF components (fibroin/sericin) intrinsically activate Wnt signaling pathway‐mediated osteogenesis, in agreement with a previous study [40]. Mechanical stimulation after implantation also potentiates TGF‐β‐ and PI3K‐Akt signaling pathway‐driven enthesis repair. Previous studies have also shown that mechanical stimulation is essential in promoting enthesis injury repair [41]. Collectively, we propose that the angle‐ply, three‐dimensionally arranged SF (i.e., silk/glass fiber yarns interwoven at $30^{\circ }$ with SF at the surface) promotes graft‐bone healing via the Wnt, TGF‐β, and PI3K‐Akt signaling pathways. Based on all the comparative transcriptomics, we conclude that the three‐dimensional porous structure and bioactive SF components of SF‐GFs can activate osteogenesis‐related signaling pathways, whereas GF components are more likely to promote biomimetic mineralization and fibrillation. Besides, the enhanced vascularization, as evidenced by CD31 immunostaining, in the SF‐GF group likely contributed to the rapid nutrient supply and stem cell recruitment, thereby accelerating the osseoligamentous healing process.

### 3.3 Enhanced Osseointegration Drives Functional Recovery

We further implanted SF, SF‐GF, and woven PET fibers into rabbit femoral tunnels under identical conditions and conducted in vivo histological analysis. μCT imaging revealed progressive infiltration of newly formed bone into the tunnel space and partial infiltration into the SF and SF‐GF scaffolds. No clear boundary was observed between the SF‐GF scaffolds and the surrounding bone tissue. In contrast, minimal new bone formation was seen around PET grafts within the femoral tunnels. Comparative analysis of bone regeneration between the SF‐containing scaffolds and the PET controls indicated that the presence of SF fibers significantly enhanced tissue formation and osseointegration. The pull‐out force measurements were comparable to the tensile strength of native ligaments, further indicating effective bone–graft integration. In clinical studies, cases where basic tissue repair does not restore the original function are very common [42], so we focus more on the recovery of motor function. Due to the enhanced interfacial bonding facilitated by bone ingrowth into SF‐GF scaffolds, rabbits implanted with SF‐GF ligaments exhibited significantly restored functional mobility, characterized by a more stable gait and longer walking distances. In contrast, rabbits with torn ACLs exhibited only limited locomotion, such as jogging, and were unable to jump.

Consistent with the μCT and biomechanical results, H&E‐stained sections confirmed substantial bone ingrowth into the SF‐GF scaffolds. Notably, a thick layer of anisotropically aligned collagen, resembling that of native bone tissue (critical for bone mineralization) [43], was observed at the interface between the SF‐GF scaffolds and host bone. Conversely, little to no bone or collagen formation was observed at the PET scaffold interfaces. In vivo immunofluorescence staining demonstrated elevated expression of osteogenesis/ligament related proteins Runx2, OCN and COL1 in SF‐containing scaffolds compared to the control group, supporting the involvement of the Wnt signaling pathway in SF‐induced osteogenesis. These findings collectively indicate that SF‐GF and SF scaffolds significantly enhance new bone formation and osteogenic activity relative to PET scaffolds, likely attributable to the bioactive nature of silk fibers.

Scaffold re‐tear rates were 75% (3/4 animals) for SF scaffolds, 25% (1/4 animals) for PET scaffolds, and 0% for SF‐GF scaffolds. The incorporation of GF imparted the SF‐GF scaffolds with a higher Young's modulus, thereby better supporting mechanical loads applied to the knee compared to SF scaffolds alone. For comparison, reported re‐tear rates in patients using current commercial ACL implants are typically around 30% [44, 45]. However, these commercial implants merely replace the native tissue, lack degradability, and do not actively promote tissue regeneration. In the in vivo ACL reconstruction model, the macroscopic structural integrity of all constructs remained intact upon retrieval. Such a delayed degradation profile is intentional and highly advantageous for load‐bearing artificial ligaments. Specifically, although silk fibroin is incorporated as a bioactive component, its highly crystalline braided structure inherently exhibits a remarkably slow and controllable degradation rate in vivo [46, 47].

While the SF‐GF scaffold holds considerable promise for ACL reconstruction, several limitations must be acknowledged. The precise mechanisms by which the graft promotes bone marrow stem cell adhesion, proliferation, and differentiation remain unclear. Future investigations will aim to elucidate the key signaling pathways involved in SF‐GF‐mediated regulation of stem cell fate. Moreover, it should be noted that this study utilized New Zealand white rabbits, a small animal model, and the observation period was relatively short. Given the differences in mechanical loading patterns and joint alignment between quadrupedal animals and the human knee, extrapolation of these findings to clinical applications requires further validation. Furthermore, while open‐field tests quantified macroscopic motor recovery, the lack of precise joint kinematic gait analyzes limits a thorough biomechanical evaluation of locomotion. Accordingly, long‐term studies in large animal models (e.g., beagle dogs or pigs) incorporating formal gait assessments are essential to comprehensively assess the translational potential of SF‐GF scaffolds. Finally, SF/GF resorption rates must align with neo‐tissue formation; in silico modeling could optimize tunability parameters.

## 4 Conclusions

In this study, we feature an innovative biomaterial design through a programmable “angle‐ply” architecture that dynamically emulates the nonlinear and viscoelastic mechanics of native ligaments, along with a quantitative structure‐property relationship established via computational modeling. Importantly, the bioactive silk fiber significantly promotes osseoligamentous integration. Although it requires further validation in long‐term studies, the design concept establishes a promising framework for future biomedical applications. In the foreseeable future, such an approach could enable AI‐assisted customization of artificial ligaments, leading to the realization of programmable, patient‐tailored artificial grafts. Furthermore, the programmability and tunable mechanics of the framework offer potential across diverse fields, including tissue engineering scaffolds, soft robotics, wearable assistive systems, flexible electronics, and aerospace composites.

Overall, this methodology provides a versatile and forward‐looking blueprint for programmable braided materials that connects patient‐specific graft design with multifunctional material engineering, offering a pathway toward adaptive, precision‐engineered biomedical constructs.

## 5 Methods

### 5.1 Materials Preparation

To produce SF‐GF scaffolds, raw SF (National Silkworm Genetic Resources Gene Bank, Chongqing, China) and S‐glass fiber (GF; Nanjing Fiberglass Research & Design Institute Co., Ltd, China) yarns were processed by combining 4 to 8 cocoons into raw fiber threads. The S‐glass fiber was manufactured with a formulation of ${\mathrm{SiO}}_{2}$, ${\mathrm{Al}}_{2}O_{3}$, and MgO [48]. This specific alkali‐free aluminosilicate composition was selected because it confers not only exceptional ultimate tensile strength and fatigue resistance but also remarkable hydrolytic stability and chemical inertness in aqueous physiological environments. The reeled fibers were twisted into yarn and used to fabricate 3D braided spacer fabric via three‐dimensional five‐directional (3D–5D) braiding technology (3DB‐R100/1C1, China). Each SF‐GF scaffold comprised 37×2 silk filaments (axial) and 51 glass filaments (braided), optimized to approximate native ACL mechanics. For the PET control fabric, filaments were produced on a pilot spinning line (Hills Inc.) using PET Type RT52 (${\mathrm{INVISTA}}^{TM}$ Resins & Fibers) with an intrinsic viscosity of 0.64. All the SF, SF‐GF and PET scaffolds were washed in a 0.15% (w/v) natural soap solution (2 h) and rinsed with distilled water to remove manufacturing residues. SF‐GF scaffolds underwent additional sericin extraction by boiling in 0.03 M ${\mathrm{Na}}_{2}{\mathrm{CO}}_{3}$ (1 h), followed by rinsing with boiling distilled water.

### 5.2 Morphological Characterization

#### 5.2.1 Scanning Electron Microscopy

SEM analysis was performed to investigate the surface morphology and the spacer structures of each group of fibers and artificial ligaments. Samples were sputter‐coated with gold (Fisons Instruments, Sputter Coater SC502, UK) prior to analysis and the micrographs were acquired at an accelerating voltage of 15 kV at different magnifications.

#### 5.2.2 Micro‐Computed Tomography

The re‐twisted microstructure of the spacer textile structures was evaluated using a high‐resolution X‐ray microtomography system (Skyscan 1072 scanner, Skyscan, Belgium). Samples were scanned for both external sheets (Top) and internal monofilaments (Bulk). SF‐GF samples were scanned at a pixel size of 11.31 μm (x/y/z) with an X‐ray source set to 41 keV and 215 μA, whereas PET fabrics were scanned at 14.71 μm (x/y/z) with 40 keV and 154 μA. Representative serial images (Top and Bulk) were thresholded using dynamic thresholds of 60–225 for SF‐GF and 59–255 for PET, determined from grayscale histograms of material density profiles. Morphometric analysis (CT Analyzer v1.5., SkyScan, Belgium) quantified porosity, mean pore size, and mean pore thickness. Three‐dimensional virtual models of the spacer structures were reconstructed, visualized, and analyzed using CT‐Vox and Data Viewer software (SkyScan, Belgium). Three specimens per material were analyzed to ensure reproducibility of qualitative and quantitative assessments.

### 5.3 Mechanical Properties

#### 5.3.1 Tensile Tests

The mechanical behavior of the spacer textile structures under quasi‐static tensile loading was evaluated using an Instron 5966 universal testing machine equipped with a 2580 series static load cell. The gauge length was 30 mm and the tensile rate was 5 mm/min, which corresponds to an initial strain rate of approximately 0.0028 $s^{-1}$. This defined strain rate allows for the evaluation of the time‐dependent nonlinear mechanics of the grafts. At least 4 replicates from each group of fibers were used for the mechanical testing. Stiffness (force per unit displacement) and modulus (stress per unit strain) were calculated from the tensile force‐displacement and stress–strain curves, respectively (Figure S6).

#### 5.3.2 Dynamic Tensile/Torsional Fatigue Tests

For dynamic tensile testing, specimens were subjected to cyclic axial loading (25–250 N) at 5 Hz using a Dynamic & Fatigue Tester (Dynamess TP5LCF, Germany). This 5 Hz loading frequency corresponds to high dynamic physiological strain rates, which is essential to verify the viscoelastic strain‐rate dependency and durability of the grafts under rapid multidirectional loading. Four independent specimens were tested for each tensile cycle group. In the dynamic torsion test, a pre‐load of 250 N was applied, followed by torsional loading at $15^{\circ }$, 4 Hz, for durations of 0, 8, and 16 h, corresponding to 0, 115,200, and 230,400 cycles (calculated as cycles = 4 Hz × 3600 s/h × time). Three replicates were tested per torsional cycle group. The test was performed using a bovine serum lubricant with a protein concentration of 20 g/L. The lubricant was replaced every 500 000 cycles, and the used serum containing wear debris was collected and stored. The ligament was cleaned and weighed after each interval to monitor mass loss.

#### 5.3.3 Elastic Recovery Performance Test

This study employed immediate elastic recovery testing to characterize the elastic recovery properties of artificial ligaments using a textile medical material strength tester (YG‐B 026G‐500). All specimens were preconditioned prior to elastic recovery testing under the following parameters: initial gauge length of 20 mm, preconditioning load of 5 N, extension speed of 120 mm/min (equivalent to a higher physiological strain rate of 10 %/s) and 3 cyclic repetitions. Following preconditioning, single‐cycle elastic recovery testing was conducted under ultimate loading conditions: maximum load of 100±15 MPa (approaching but significantly higher human ACL rupture strength), with identical loading/unloading speeds of 120 mm/min and 1 s dwell time. Three replicates per sample were tested, with mean values and standard deviations calculated.

#### 5.3.4 Creep Response Tests

The time‐dependent creep response of the braided ligaments was investigated to evaluate their resistance to long‐term mechanical laxity. To determine the effect of long‐term cyclic loading on viscoelastic stability, creep tests were performed on both pre‐fatigue specimens and specimens that had undergone dynamic fatigue loading. Specimens with an initial gauge length of 30 mm were immersed in a phosphate‐buffered saline (PBS) bath at 37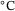 and subjected to a constant physiological tensile load of 250 N [49]. The resulting elongation (creep strain) was continuously recorded for 900 s. The primary and secondary creep behaviors before and after fatigue were analyzed and compared with the known viscoelastic creep responses of the native ACL.

#### 5.3.5 Finite Element Analysis

Textile composites exhibit hierarchical architectures and complex fiber‐level behaviors. To accurately represent the geometry of our three‐dimensional, five‐directional hybrid weave, we adopt the fiber‐scale modeling approach of Ghaedsharaf et al. [50] In order to capture inter‐fiber friction, the General Contact algorithm in Abaqus/Explicit Dynamics is used to define tangential contact with a friction coefficient of 0.30 ‐ an empirically validated value that faithfully reproduces observed fiber interactions [51]. To mitigate computational expense, periodic boundary conditions are imposed on a representative unit cell, reflecting the material's inherent periodicity along the weave axis. Finally, because the composite's mechanical response is dominated by tensile behavior, the fibers are idealized as truss elements.

### 5.4 In Vitro Cell Culture, Transcriptomics, and Osteogenic Assays

#### 5.4.1 Cytocompatibility of the Textile Scaffolds

SF‐GF scaffolds were incubated in α‐MEM (10% fetal bovine serum [FBS], 1% penicillin/streptomycin) at $37^{\circ }C$, 5% ${\mathrm{CO}}_{2}$, and 95% humidity for 24 h to generate conditioned extracts. Conditioned extracts were prepared at 0.2 g/mL ratio, according to ISO 10993‐5 guidelines [52]. Normal mouse fibroblasts (L929 cells) were incubated with scaffold extracts for 24, 96 and 168 h. Cell viability was assessed using the CCK‐8 assay (Beyotime, China), measuring absorbance at 450 nm. Six biological replicates were tested per group.

#### 5.4.2 Osteogenic Differentiation of MC3T3‐E1 Cells in the Scaffolds

MC3T3‐E1 cells (BNCC33398, BeNa Culture Collection, China) were cultured in EMEM complete culture medium (containing 10% FBS) (BNCC338137, BeNa Culture Collection,China) at $37^{\circ }C$ with 5% ${\mathrm{CO}}_{2}$. SF‐GF and PET scaffolds were cut into cylinders (3 mm diameter × 5 mm thickness), sterilized with ethylene oxide, and hydrated overnight in PBS. MC3T3‐E1 cells were trypsinized, seeded onto three different artificial ligament scaffolds at a density of $5\times 10^{4}$ cells/scaffold and cultured for 7 days. The morphology of cells cultured on the scaffolds was analyzed using SEM. Cell‐containing samples were first dehydrated following a graded ethanol series, consisting of 15‐min incubation steps in solutions with increasing ethanol concentrations of 50%, 60%, 70%, 80%, 90%, 95%, and 100%. Afterward, samples were briefly air dried and incubated in hexamethyldisilazane (HMDS) for 15 min (Sigma–Aldrich). HMDS was then removed and samples were left to air dry overnight.

To explore the potential molecular mechanisms underlying osteogenic effects of the ligament, MC3T3‐E1 cells were directly seeded onto SF, SF‐GF, and PET spacer scaffolds. For in vitro experiments, MC3T3‐E1 cells ($1\times 10^{6}$ cells) were cultured on PET, SF‐GF, and SF scaffolds for subsequent transcriptomic analysis. After the culture period, cells were lysed in Trizol reagent (Beyotime Biotechnology, cat. no. R0016), and total RNA was extracted according to the manufacturer's protocol. RNA samples were stored at –$80^{\circ }C$ until further processing. For library preparation, 500 ng of RNA per sample was used as input. Ribosomal RNA was depleted using the Ribo‐off rRNA Depletion Kit (Vazyme, N406‐02), and sequencing libraries were constructed with the VAHTS Universal V6 RNA‐seq Library Prep Kit for Illumina (Vazyme, NR604) following the manufacturer's recommendations. Index codes were added to allow for multiplexed sequencing. Transcriptome sequencing was performed on an Illumina HiSeq X10 platform (Illumina). Gene expression was quantified using the FPKM (fragments per kilobase of transcript per million mapped reads) metric. For downstream analysis, transcripts with an FPKM value >1 were retained to exclude low‐abundance transcripts.

#### 5.4.3 Immunodetection of Bone‐Specific Proteins

MC3T3‐E1 cells on scaffolds were fixed in 4% paraformaldehyde (15 min), permeabilized with 0.1% Triton X‐100 (20 min) and blocked with 3% bovine serum albumin (BSA; 30 min). Primary antibodies included: OCN antibody (Santa Cruz Biotechnology, sc‐390877, 1:100); COL1A antibody (Cell Signaling Technology, 72026T, 1:200); Osteopontin (OPN) antibody (Cell Signaling Technology, 27927T, 1:1600). Following incubation with Goat Anti‐Mouse IgG H&L (Alexa Fluorö 488) (20 min, Abcam, ab150113) counterstaining with DAPI (10 min; Solarbio, China), images were acquired using TCS SP8 STED 3X microscope (Leica, Germany).

### 5.5 In Vivo ACL Reconstruction, Kinematic, and Histological Evaluations

Animal experiments were approved by the Institutional Animal Care and Use Committee at Nanjing Jinling Hospital (number 2023JLHGZRDWLS‐ 00018). All animals were housed at the animal care laboratory at the Hospital of Pharmacy in accordance with the regulations set by the National Institutes of Health.

#### 5.5.1 Biosafety Evaluation of SF‐GF Scaffolds In Vivo

To explore the immune response to potential wear debris generated from glass fibers, we previously tested wear debris collected during dynamic fatigue testing of silk‐glass artificial ligaments. The harvested wear particles were subcutaneously implanted in 8‐week‐old male Sprague–Dawley (SD) rats. Tissue samples were harvested at 4 and 12 weeks after implantation for histological evaluation using Masson's trichrome and H&E staining of skeletal muscle around the implantation site. As a supplement to the histological evaluation, concentrations of inflammatory factors such as interleukin‐1 (IL‐1) and tumor necrosis factor‐α (TNF‐α) were measured after implantation.

#### 5.5.2 ACL Reconstruction on New Zealand White Rabbits

To evaluate the biointegration of SF‐GFs with native bones, SF‐GF grafts were implanted in a standard rabbit ACL reconstruction model. After a 2‐week quarantine period, 24 skeletally mature male New Zealand White rabbits (3.0–3.5 kg) were randomized into three groups (n=4/group): PET graft group; SF‐GF graft group; SF graft group (defect‐only). The PET, SF‐GF, and SF grafts shared identical three‐dimensional, five‐directional structures (3 mm diameter × 5.0 cm length), verified via microscopy (diameter) and caliper measurements (length). Rabbits were anesthetized (3% pentobarbital sodium, 30 mg/kg IV), positioned supine, and prepared with 2.5% iodophor. A 3 cm medial parapatellar incision exposed the knee joint. The native ACL was transected midsubstance, and ACL discontinuity was confirmed via positive Lachman test. Femoral and tibial bone tunnels (3.0 mm diameter) were drilled using a Kirschner wire. Grafts were secured with PDS II guide wire (Ethicon) and sutured to periosteum (No. 4‐0 Ethibond) at femoral/tibial ends under tension in the posterior Lachman position. Post‐implantation, joints were irrigated, cyclically loaded, and closed in layers. Rabbits received postoperative care: prophylactic penicillin (800 000 IU/day IM × 3 days) and daily aseptic wound cleaning.

#### 5.5.3 Motor Function Assessment

For the rabbit open‐field experiment, homemade cages were assembled into a 3.5 × 3.5 m square paddock. A camera was fixed 5 m above the center of the paddock and connected to a computer running the Smart 3.0 system. Rabbits were allowed to move freely for 10 min. Distance traveled, maximum speed, average speed, rest time, and other changes in locomotor ability during free and pressured movement were recorded.

#### 5.5.4 μCT Evaluation and Analysis

At 4 and 12 weeks after implantation, bone regeneration was evaluated by ex vivo μCT (Y. XLON Cheetah, Germany; resolution: 15.3 μm, voltage: 80 kV, current: 70 μA). Graft‐tissue blocks were immersion‐fixed in 4% formaldehyde for 14 days, subjected to scanning, and reconstructed using VGStudio MAX 3.0 (Volume Graphics, Germany). Bone volume (BV), total scaffold volume (TV), and BV/TV ratios were calculated.

#### 5.5.5 Histology and Immunofluorescence Staining

Following fixation in 4% paraformaldehyde (48 h, $4^{\circ }C$) and decalcification for 4 weeks, histological examination proceeded. The samples were sectioned to 8 μm thickness, and stained with H&E, VG and Masson's trichrome (collagen visualization). Histological scoring was performed by two blinded observers using a semi‐quantitative scale (0: none, 1: mild, 2: moderate, 3: marked) for inflammation (H&E), bone regeneration (H&E/VG), and vascularization (Masson's trichrome) by software Image J.

For the immunofluorescence staining, the graft‐bone samples were incubated with Triton X‐100/10% BSA (0.2%, in PBS) for permeabilization and blocking. The sections were then incubated with primary antibodies: Runx2 (Servicebio, 1:200, Rab# [GB115631]); OCN (Servicebio, 1:200, Rab# [GB11233]). Secondary antibodies (Cy3‐labeled, 1:500) and DAPI (Sigma–Aldrich) were applied. Images were captured using a fluorescence microscope (NIKON ECLIPSE E100, Japan). Protein expression was quantified via ImageJ (NIH, USA) using integrated density thresholds. For long‐term in vivo safety evaluations, rabbits implanted with SF‐GFs for different durations were killed. The joint tissues containing SF‐GFs were fixed as above. Sections were stained using an H&E staining kit (Servicebio, G1005) and a Masson's trichrome staining kit (Servicebio, G1006). As supplement to long‐term in vivo safety, fresh organs of synovium, liver, and kidney were fixed with 4% w/v paraformaldehyde for 48 h at $4^{\circ }C$ and embedded in paraffin. Sections were cut to a thickness of 8 μm and subjected to H&E, VG and Masson staining according to standard protocols and observed using an optical microscope (BX53, Olympus, Japan).

All numerical data are expressed as mean ± standard deviation (SD). Statistical analyzes were performed using GraphPad Prism 5.0 (GraphPad Software, USA). Static mechanical properties (e.g., stiffness, ultimate load) were compared between groups using unpaired Student's t‐tests (two groups) or one‐way ANOVA with Tukey's post‐hoc test (≥3 groups). Biological assays (e.g., CCK‐8, COL1A activity, RT‐PCR) were analyzed using the Kruskal–Wallis test (non‐parametric) followed by Dunn's multiple comparisons test. Cell differentiation studies (calcium deposition, gene expression) involved three independent experiments, with four replicates per group at each time point. Statistical significance was defined as *p<0.05, **p<0.01, ***p<0.001.

## Author Contributions

**Changbo Lu**: experiments conduction, formal analysis, writing – original draft preparation. **Daming Liu**: experiments conduction, formal analysis. **Haoxin Zhang**: experiments conduction, software, writing – original draft preparation. **Zhiwei Qiao**: data curation, validation. **Qiuming Gao**: writing – review & editing. **Jia Meng**: experiments conduction. **Hongyin Yue**: experiments conduction. **Songkai Li**: writing – original draft preparation. **Shuo Zhang**: resources, data curation. **Tao Yuan**: software. **Yun Xue**: writing – original draft preparation. **Hui Jiang**: software. **Zhenguo Liu**: experiments conduction. **Yibo Wang**: conceptualization, experiments conduction, formal analysis. **Nirong Bao**: conceptualization, writing – review & editing. **Yuli Chen**: conceptualization, writing – review & editing. **Xiaojiang Yang**: funding acquisition, conceptualization, writing – review & editing.

## Conflicts of Interest

The authors declare no conflicts of interest.

## Supporting information


**Supporting File 1**: advs78414‐sup‐0001‐SuppMat.pdf.

**Supporting File 2**: advs78414‐sup‐0002‐Video1.mp4.

## Data Availability

The data that supports the findings of this study are available in the supplementary material of this article.
