# Supplementary material for: A Programmable Braided Silk‐Glass Fiber Ligament Promoting Rapid Motoric Recovery
Source: Adv Sci (Weinh). 2026 Oct 29:e78414. Online ahead of print. doi: 10.1002/advs.78414 (PMC13720269; doi:10.1002/advs.78414)
Supplement: Supplementary file 1 [file ADVS-9999-e78414-s001.pdf]

Supplementary Materials

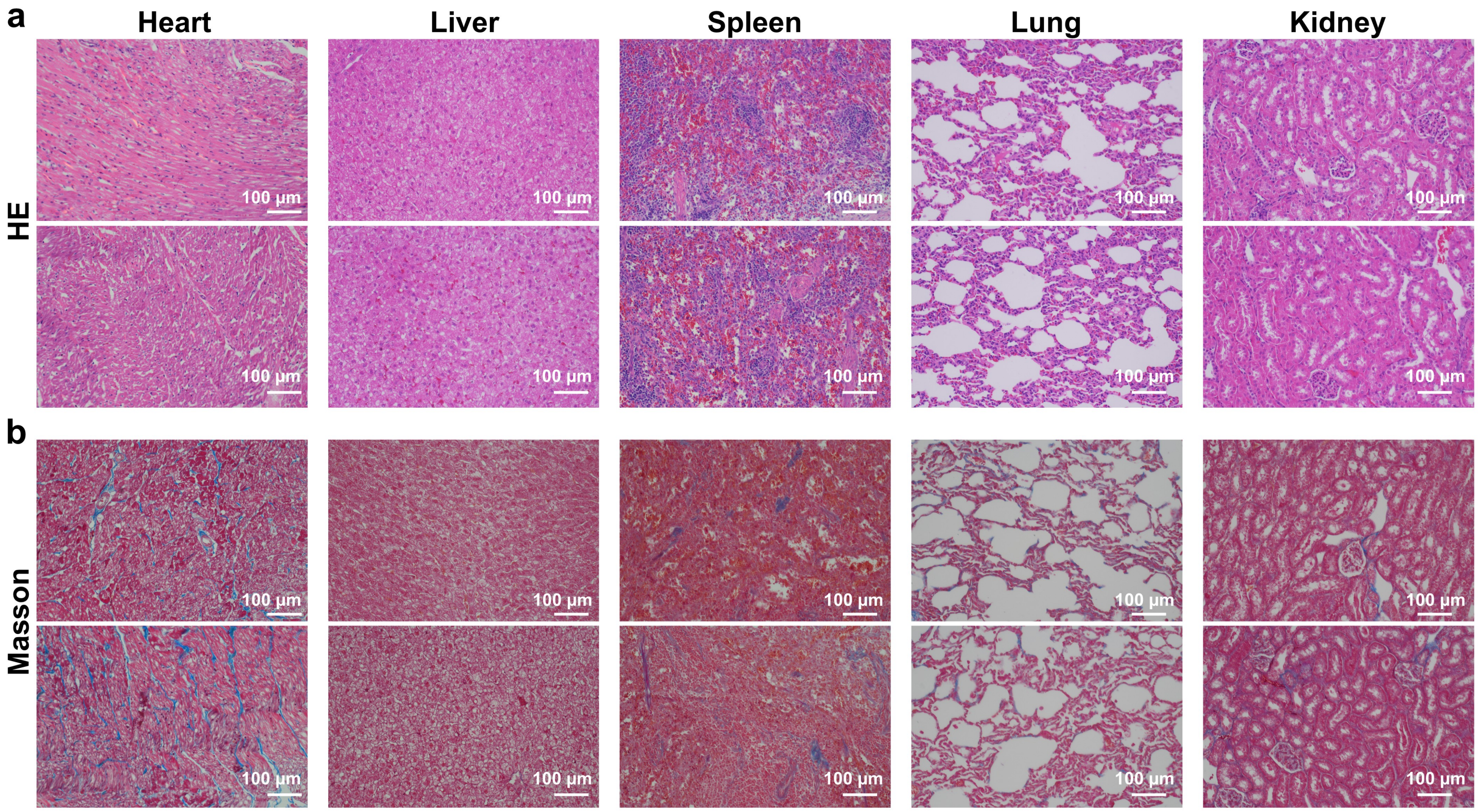

Figure S1. **Histological assessment of SF-GF graft long-term safety in vivo. a)** H&E sections of cardiac muscle, liver, spleen, lung and kidney after 12 weeks of SF-GF implantation reveal no evidence of residual GF fragments. Control: non-implanted rabbits. **b)** Masson's trichrome-stained tissue sections of cardiac muscle, liver, spleen, lung and kidney 12 weeks post-SF-GF implantation reveal no significant fibrotic collagen deposition or peribronchial lymphocyte accumulation across examined tissues. Control: non-implanted rabbits. Histochemical interpretation: red denotes cytoplasm, muscle fibers, and erythrocytes; blue indicates collagen.

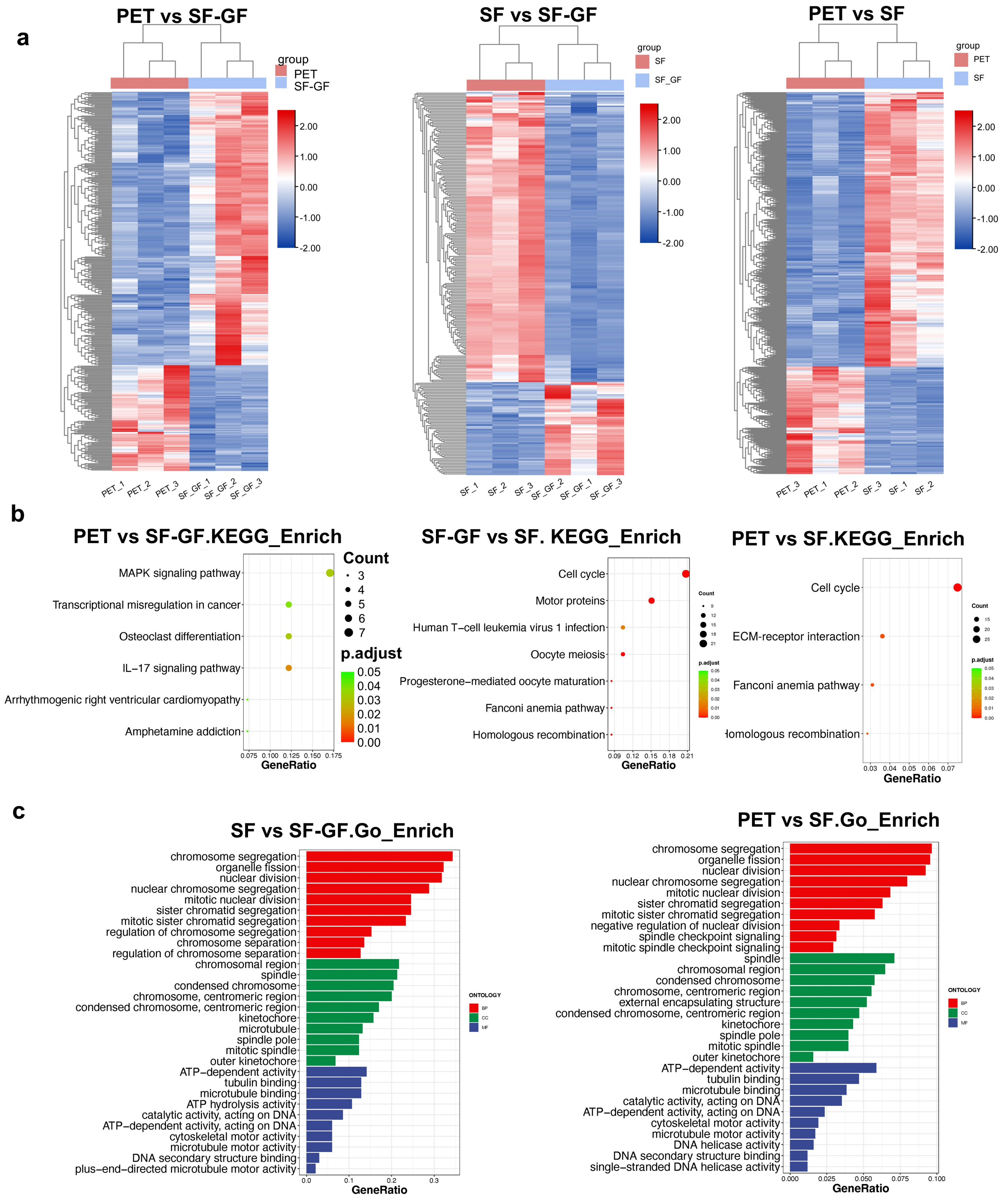

Figure S2. **SF-GF promotes osteogenesis in vitro via signalling pathways.** **a)** A clustered heatmap of the transcriptomic analysis of MC3T3-E1 cells shows upregulated osteogenesis-related signalling pathways for different pairwise comparisons. **b-c)** GO and KEGG enrichment based on transcriptomic analysis of MC3T3-E1 cells shows upregulated osteogenesis-related signalling pathways for different pairwise comparisons (SF component: PET vs SF; GF component: SF-GF vs SF; SF and GF component: PET vs SF-GF).

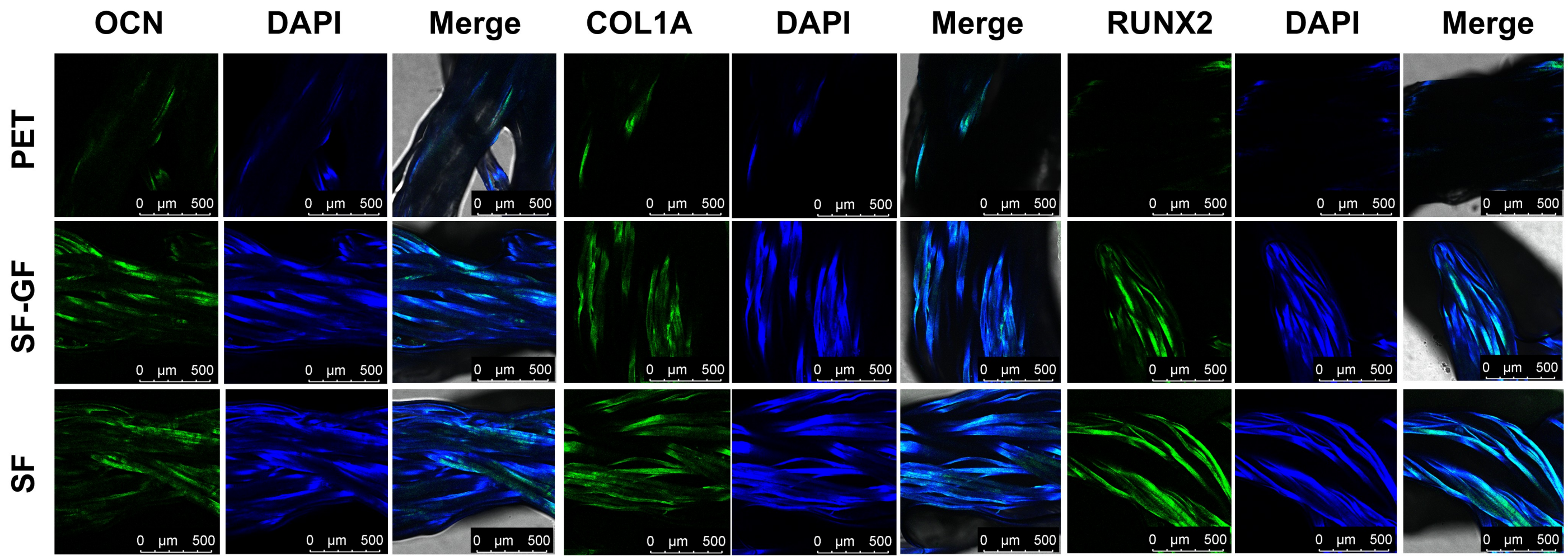

Figure S3. **Viability and morphology of rBMSCs cultured on scaffold segments (immunofluorescence)**. Targeted osteogenesis/osseoligamentous-related proteins (OCN, COL1A1, Runx2) were stained green. Cell nuclei were stained blue.

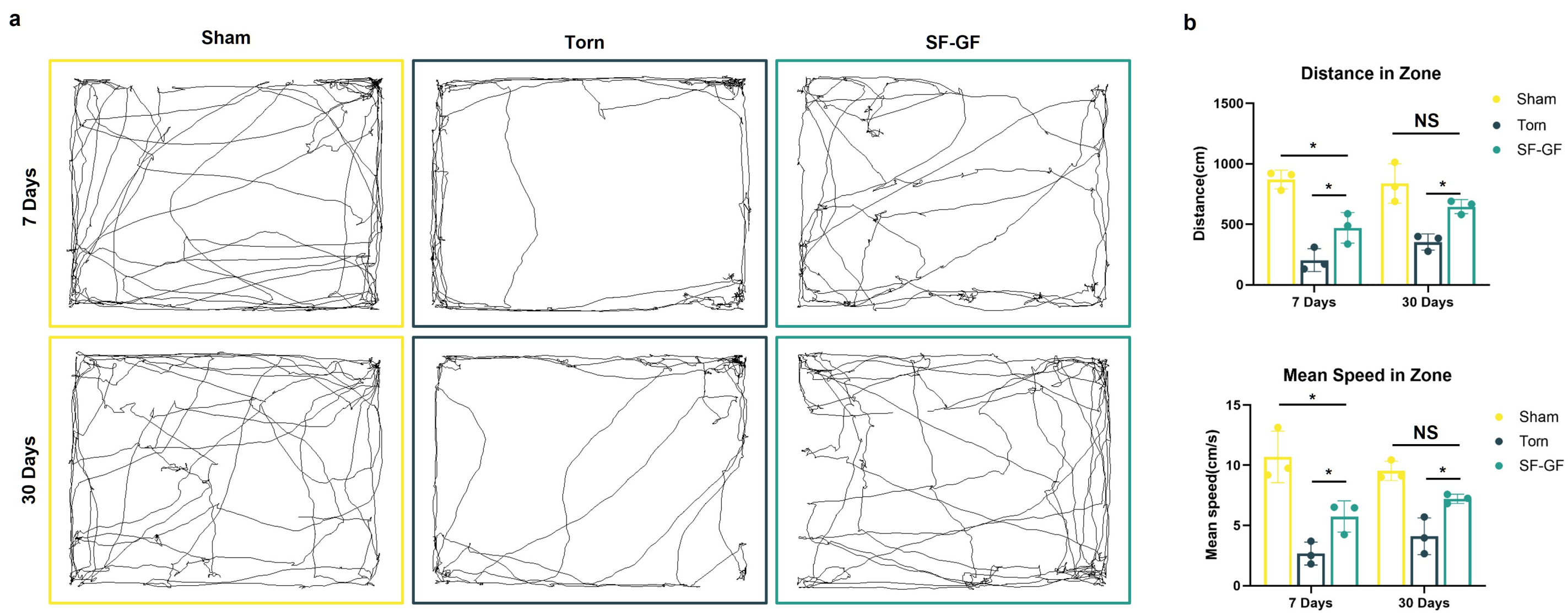

Figure S4. **Open-field tests. a)** Diagrams of open-field experiments in rabbits, with trajectories reflecting the paths traveled by the rabbits over a defined period. Detection time points were 7 days and 1 month. **b)** Data are presented as mean ± SD. The statistical significance of the differences between various treatments was determined by one-way ANOVA with Bonferroni post-test (n = 3, *p<0.05, **p<0.01, ***p<0.005).

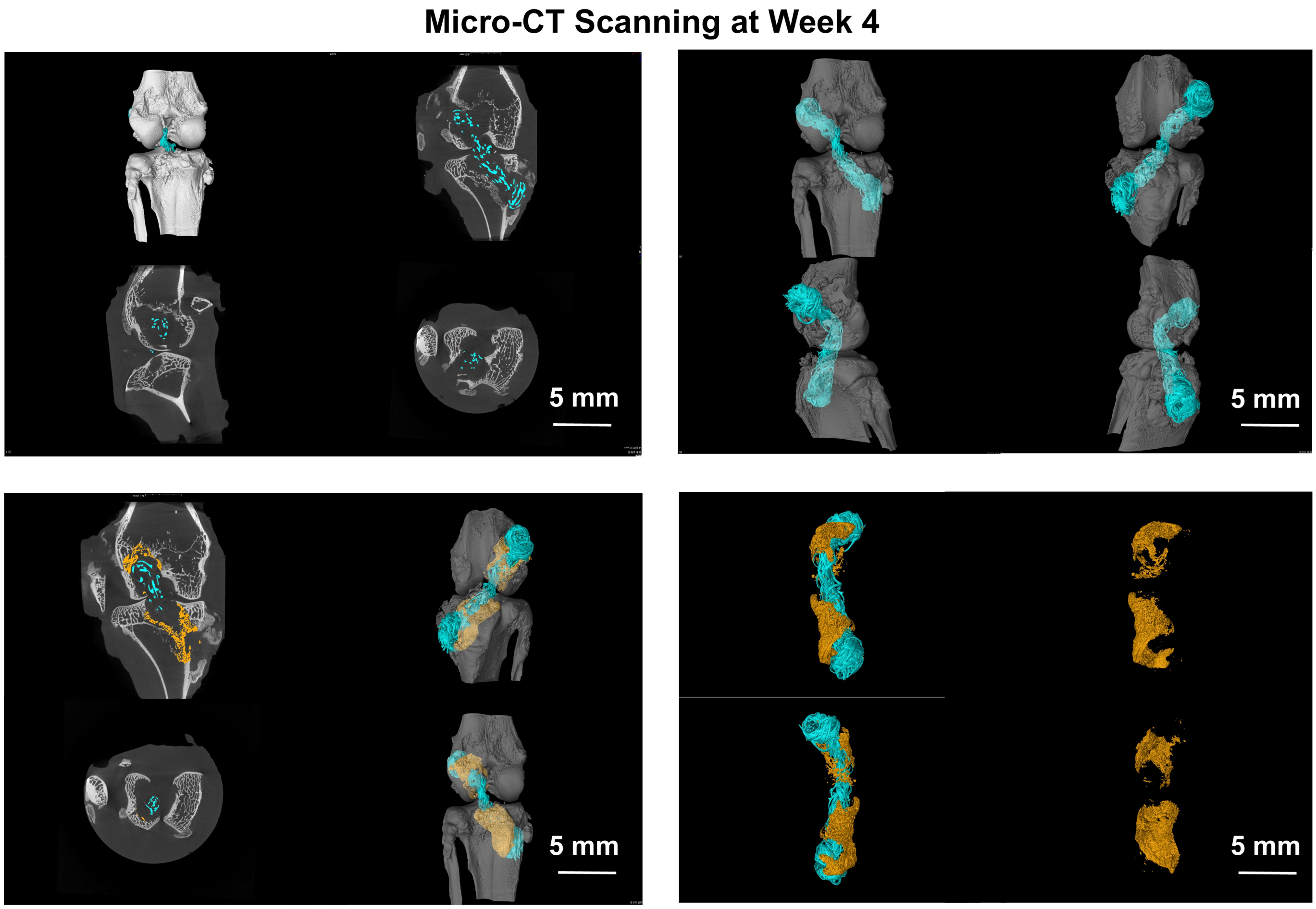

Figure S5. **µCT 3D reconstruction of the graftbone interface from different viewpoints**: new bone in yellow and ligament in blue.

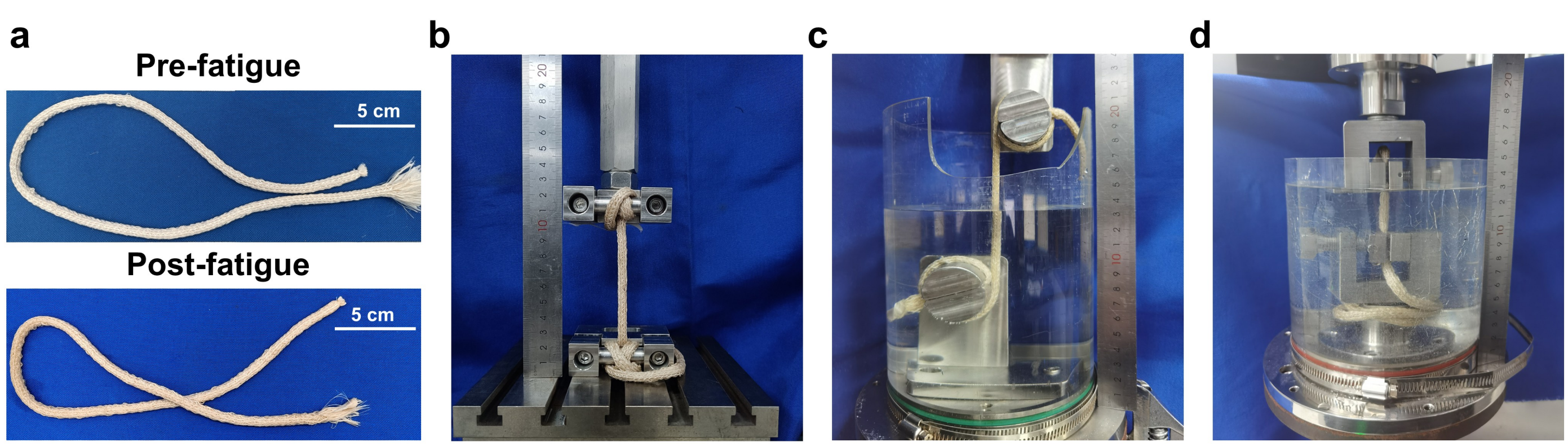

Figure S6. **Test facilities for performing mechanical tests of the artificial ligament specimens. a)** Pre-fatigue and post-fatigue of the artificial ligaments. **b)** The quasi-static tensile test facility with a Dynacell loadcell of 10 kN capacity. **c)** The dynamic tensile test facility. **d)** The dynamic tensile-torsional test facility.

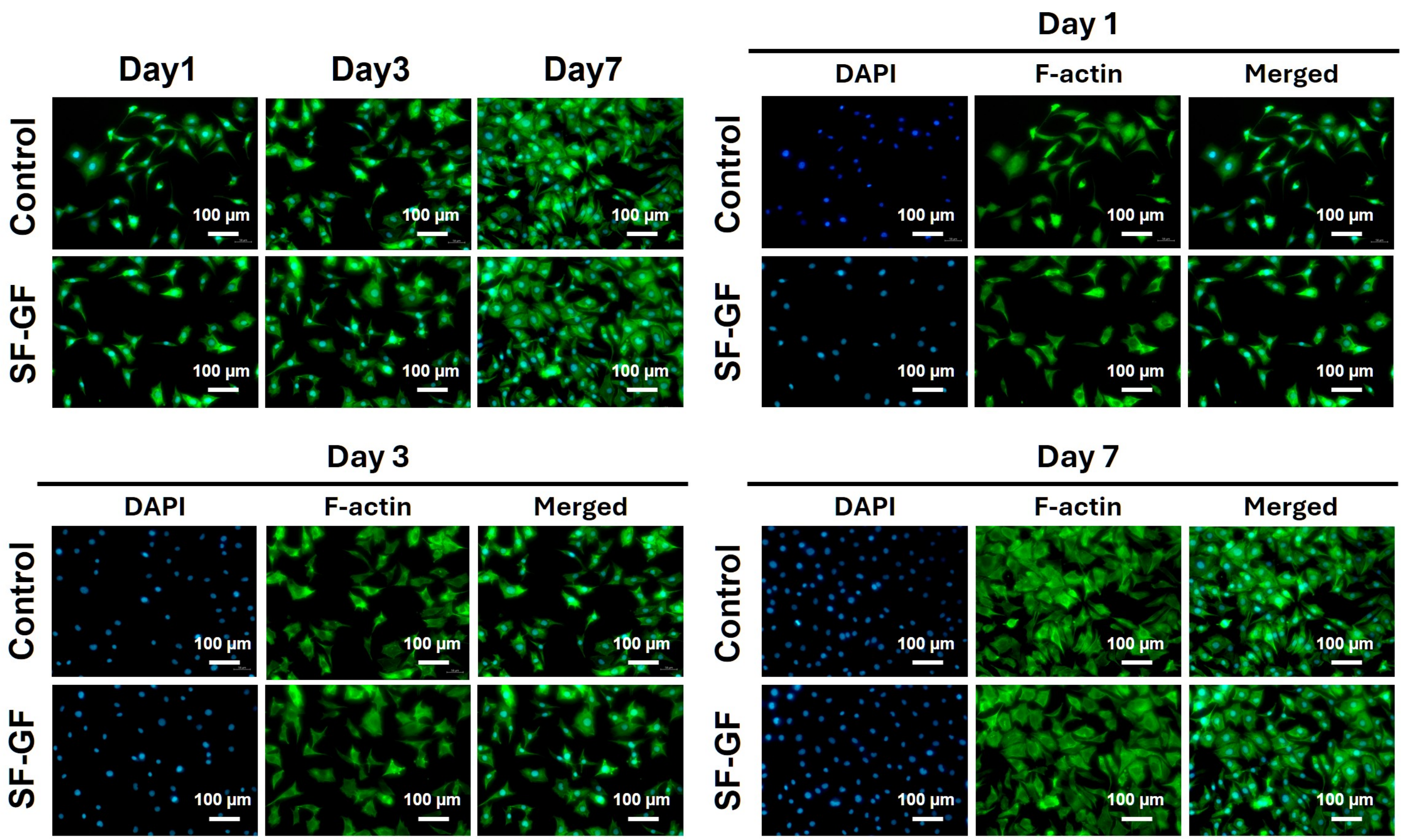

Figure S7. **Proliferation of L929 cells in SF-GF graft extracts and α-MEM on days 1, 3, and 7 (blue: nuclei; green: F-actin).**

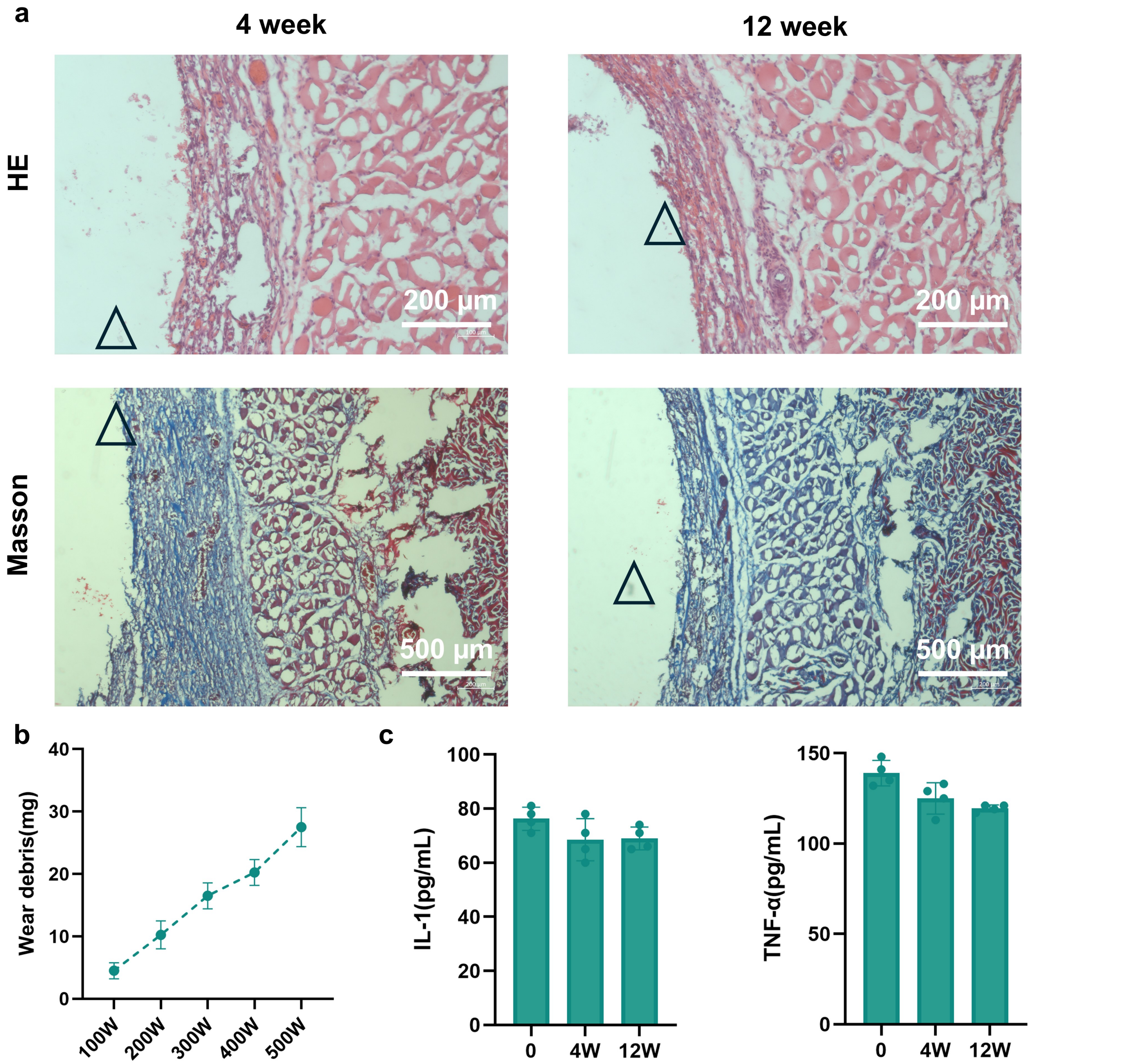

Figure S8. **Long-term biosafety evaluations of SF-GF grafts.** **a)** H&E and Masson staining show that GF debris (triangle) integrates well with skeletal muscle and does not elicit inflammatory reactions after implantation in rabbits for 12 weeks. **b)** Collected wear debris during dynamic fatigue tests. **c)** Interleukin-1 (IL-1) and tumour-necrosis factor-α (TNF-α) concentrations varied within normal physiological ranges. $n = 4$ biologically independent experiments for each group. All data are expressed as mean $\pm$ s.d.
